# Kv11 (*ether‐à‐go‐go*‐related gene) voltage‐dependent K^+^ channels promote resonance and oscillation of subthreshold membrane potentials

**DOI:** 10.1113/JP280342

**Published:** 2020-11-18

**Authors:** Toshinori Matsuoka, Miwako Yamasaki, Manabu Abe, Yukiko Matsuda, Hiroyuki Morino, Hideshi Kawakami, Kenji Sakimura, Masahiko Watanabe, Kouichi Hashimoto

**Affiliations:** ^1^ Department of Neurophysiology Graduate School of Biomedical and Health Sciences Hiroshima University Hiroshima Japan; ^2^ Department of Anatomy Faculty of Medicine Hokkaido University Sapporo Japan; ^3^ Department of Animal Model Development Brain Research Institute Niigata University Niigata Japan; ^4^ Department of Epidemiology Research Institute for Radiation Biology and Medicine Hiroshima University Hiroshima Japan

**Keywords:** hyperpolarization‐activated cyclic nucleotide‐gated channel 1 (HCN1), Kv11 (*ether‐à‐go‐go*‐related gene), resonance

## Abstract

**Key points:**

- Some ion channels are known to behave as inductors and make up the parallel resonant circuit in the plasma membrane of neurons, which enables neurons to respond to current inputs with a specific frequency (so‐called ‘resonant properties’).
- Here, we report that heterologous expression of mouse Kv11 voltage‐dependent K^+^ channels generate resonance and oscillation at depolarized membrane potentials in HEK293 cells; expressions of individual Kv11 subtypes generate resonance and oscillation with different frequency properties.
- Kv11.3‐expressing HEK293 cells exhibited transient conductance changes that opposed the current changes induced by voltage steps; this probably enables Kv11 channels to behave like an inductor.
- The resonance and oscillation of inferior olivary neurons were impaired at the resting membrane potential in Kv11.3 knockout mice.
- This study helps to elucidate basic ion channel properties that are crucial for the frequency responses of neurons.

**Abstract:**

The plasma membranes of some neurons preferentially respond to current inputs with a specific frequency, and output as large voltage changes. This property is called resonance, and is thought to be mediated by ion channels that show inductor‐like behaviour. However, details of the candidate ion channels remain unclear. In this study, we mainly focused on the functional roles of Kv11 potassium (K^+^) channels, encoded by *ether‐á‐go‐go*‐related genes, in resonance in mouse inferior olivary (IO) neurons. We transfected HEK293 cells with long or short splice variants of Kv11.1 (Merg1a and Merg1b) or Kv11.3, and examined membrane properties using whole‐cell recording. Transfection with Kv11 channels reproduced resonance at membrane potentials depolarized from the resting state. Frequency ranges of Kv11.3‐, Kv11.1(Merg1b)‐ and Kv11.1(Merg1a)‐expressing cells were 2–6 Hz, 2–4 Hz, and 0.6–0.8 Hz, respectively. Responses of Kv11.3 currents to step voltage changes were essentially similar to those of inductor currents in the resistor–inductor–capacitor circuit. Furthermore, Kv11 transfections generated membrane potential oscillations. We also confirmed the contribution of HCN1 channels as a major mediator of resonance at more hyperpolarized potentials by transfection into HEK293 cells. The Kv11 current kinetics and properties of Kv11‐dependent resonance suggested that Kv11.3 mediated resonance in IO neurons. This finding was confirmed by the impairment of resonance and oscillation at –30 to –60 mV in *Kcnh7* (Kv11.3) knockout mice. These results suggest that Kv11 channels have important roles in inducing frequency‐dependent responses in a subtype‐dependent manner from resting to depolarized membrane potentials.

## Key points

- Some ion channels are known to behave as inductors and make up the parallel resonant circuit in the plasma membrane of neurons, which enables neurons to respond to current inputs with a specific frequency (so‐called ‘resonant properties’).
- Here, we report that heterologous expression of mouse Kv11 voltage‐dependent K^+^ channels generate resonance and oscillation at depolarized membrane potentials in HEK293 cells; expressions of individual Kv11 subtypes generate resonance and oscillation with different frequency properties.
- Kv11.3‐expressing HEK293 cells exhibited transient conductance changes that opposed the current changes induced by voltage steps; this probably enables Kv11 channels to behave like an inductor.
- The resonance and oscillation of inferior olivary neurons were impaired at the resting membrane potential in Kv11.3 knockout mice.
- This study helps to elucidate basic ion channel properties that are crucial for the frequency responses of neurons.

## Introduction

In some neurons, current inputs with a specific frequency are selectively output as large voltage responses (Puil *et al*. 1986; Llinas, 1988; Hutcheon & Yarom, 2000). This property is called ‘resonance’ and is thought to be caused by frequency‐dependent impedance changes of the neuronal membrane. The neuronal membrane is electrically equivalent to a parallel resistor–capacitor (RC) circuit. Additionally, some neuronal membranes have ion channels that show inductor‐like behaviours, and comprise circuits comparable to the parallel resistor–inductor–capacitor (RLC) circuit (Puil *et al*. 1986; Hutcheon & Yarom, 2000; Erchova *et al*. 2004; Narayanan & Johnston, 2008). In this circuit, impedance at higher input current frequency range is attenuated by membrane capacitors. Meanwhile, impedance at lower frequency range is thought to be attenuated by ion channels that behave like an inductor (resonating conductance) (Hutcheon & Yarom, 2000). Electrical resonance‐like responses that occur in the circuit enhance impedance around a specific frequency, resulting in enhanced voltage output at a specific input current frequency. In neurons, the resonance is known to be further enhanced by activation of other ion channels (amplifying conductance) (Hutcheon & Yarom, 2000). Several ion channels have been suggested as candidates (Hutcheon & Yarom, 2000; Wang, 2010; Hashimoto, 2020), but detailed molecular biological information about these ion channels, such as subtypes, remains largely unclear in many systems.

Neurons in the inferior olive (IO) project climbing fibres to the cerebellar cortex and form strong excitatory synapses on Purkinje cells (Ito, 2012). IO neurons exhibit clear resonance and subthreshold membrane potential oscillation (STO) (Benardo & Foster, 1986; Bleasel & Pettigrew, 1992; Lampl & Yarom, 1997; Khosrovani *et al*. 2007). We have previously reported that this resonance is dependent on the hyperpolarization‐activated cyclic nucleotide‐gated channel 1 (HCN1) and the Cav3.1 T‐type voltage‐dependent Ca^2+^ channel (Matsumoto‐Makidono *et al*. 2016). In that report, we also found that E‐4031‐sensitive ion channels were also involved in resonance. E‐4031 is known to be a blocker for Kv11 voltage‐dependent K^+^ channels (*ether‐à‐go‐go*‐related gene (ERG)), but details of the candidate ion channel remain largely unclear.

Kv11 channels are part of the KCNH family, and are encoded by three genes: *KCNH2* (ERG1, Kv11.1), *KCNH6* (ERG2, Kv11.2) and *KCNH7* (ERG3, Kv11.3) (Perry & Sanguinetti, 2010; Gustina & Trudeau, 2012; Morais‐Cabral & Robertson, 2015; Bauer & Schwarz, 2018). The KCNH family shares structural similarities with HCN and cyclic nucleotide‐gated (CNG) channels, which are part of the cyclic nucleotide binding domains (CNBD) family of cation channels (James & Zagotta, 2018). Kv11 shows fast inactivation and recovery from inactivation, compared with its activation and deactivation (Perry & Sanguinetti, 2010; Gustina & Trudeau, 2012; Bauer & Schwarz, 2018), which makes Kv11 evoke greater conductance by hyperpolarization from depolarized potentials. It has been reported that Kv11 regulates the resting membrane potential and action potential firing in neuronal and non‐neuronal cells (Akbarali *et al*. 1999; Schafer *et al*. 1999; Overholt *et al*. 2000; Rosati *et al*. 2000; Sacco *et al*. 2003; Niculescu *et al*. 2013). Pathophysiological analyses have demonstrated that mutations in *KCNH2* are linked to type II long QT syndrome (Sanguinetti *et al*. 1995). Furthermore, *KCNH7* has been identified as one of the causative genes for bipolar spectrum disorders (Kuo *et al*. 2014; Strauss *et al*. 2014). However, the roles of Kv11 in membrane potential resonance and oscillation are still unknown.

In this study, we examined the functional roles of Kv11 in resonance and membrane potential oscillation. We transfected mouse Kv11.1 or Kv11.3, which are expressed in the IO, into human embryonic kidney 293 (HEK293) cells, and examined their functional roles in resonance. We also confirmed the effects of transfection of HCN1, which mediates resonance in a wide range of brain regions, on resonance at hyperpolarized potentials. Finally, the distribution and functional roles of Kv11.3 on resonance were examined in IO neurons, mainly using *Kcnh7* knockout (KO) mice.

## Methods

### Ethical approval

All animal experiments were performed in accordance with the guidelines of the experimental animal ethics committees (No. A20‐51) and the biosafety committee for living modified organisms (No. 2020‐67) of Hiroshima University, Hokkaido University and Niigata University. C57BL/6J mice were obtained from CREA Japan (Tokyo, Japan). In this study, we generated *Kcnh7* KO mice (see Fig. 9). We maintained all mice in specific‐pathogen‐free conditions on a 12 h light/dark cycle (lights off at 20.00 h) with free access to food and water. Mice were randomly picked from littermates. For electrophysiology, coronal brain slices were prepared from C57BL/6J and *Kcnh7* KO littermates. Mice were placed in the chamber and then the CO_2_ level was increased. After losing consciousness, mice were decapitated. For fluorescent *in situ* hybridization, C57BL/6J mice were deeply anaesthetized by pentobarbital (100 μg g^−1^ of body weight, i.p.) and decapitated. For immunofluorescence, C57BL/6J and *Kcnh7* KO littermates were deeply anaesthetized by pentobarbital (100 μg g^−1^ of body weight, i.p.) and transcardially fixed with 4% paraformaldehyde in 0.1 m PB (pH 7.2). For viral vector injection, C57BL/6J mouse pups were anaesthetized by inhalation of vaporized isoflurane (4% induction, 2% maintenance). The mice were finally processed for immunofluorescence. To transfer the blastocysts into the uteri, pseudopregnant surrogate CD‐1 mice were anaesthetized by inhalation of vaporized isoflurane (2−3%). Five of six wild‐type mice and three of four *Kcnh7* KO mice were analysed by researchers blinded to their genotypes for the data shown in Fig. 10 *A*–*C*. Furthermore, all mice were analysed by researchers blinded to their genotypes for the data shown in Fig. 10 *D*–*H*. All mice were analysed in unblinded conditions for the data in Fig. 10 *I* and *J*. One of three *Kcnh7* KO mice and one wild‐type mice were analysed in blinded conditions in Fig. 10 *K* and *L*. Male or female C57BL/6J mice and *Kcnh7* KO mice at postnatal day (P)14–P29 were used for all experiments. There was no difference between sexes in the subthreshold membrane properties of IO neurons. The investigators understand the ethical principles under which *The Journal of Physiology* operates and certify that our work complies with the animal ethics checklist.

### Generation of *Kcnh*7 KO mice

C57BL/6 BAC genomic clones, RP23‐35P3 and 415D17, containing the Kv11.3 gene (*Kcnh7*) were isolated from an RP23 mouse genomic BAC library (Advanced GenoTechs, Tsukuba, Japan). To construct the *Kcnh7* targeting vector, a 0.8 kb DNA fragment carrying exon 6 of *Kcnh7* was amplified by polymerase chain reaction (PCR) and inserted into a middle entry clone (pDME‐1). In this clone, a DNA fragment of pgk promoter‐driven neomycin resistant gene‐p(A) (pgk‐Neo) flanked by two frt sites and loxP sequences was located at the site 360 bp upstream of exon 6, while the other loxP sequence was placed at the site 232 bp downstream of exon 6. The 3.4 kb upstream and 6.7 kb downstream homologous genomic DNA fragments were retrieved from the BAC clone using the Quick and Easy BAC Modification Kit (Gene Bridges, Dresden, Germany), and then subcloned to a 5′ entry clone (pD5UE‐2) and a 3′ entry clone (pD3DE‐2), respectively. For targeting vector assembly, the three entry clones were recombined to a destination vector plasmid (pDEST‐DT; containing a diphtheria toxin gene for negative selection) using a MultiSite Gateway Three‐Fragment Vector Construction Kit (Invitrogen, Waltham, MA, USA). To establish the *Kcnh7*‐flox mouse line, we electroporated the linearized targeting vector into the C57BL/6N‐derived ES line, RENKA, and then selected recombinant clones under medium containing 175 μg ml^−1^ G418. Culture of ES cells was performed as described previously (Mishina & Sakimura, 2007). The targeted clones were confirmed by Southern blot analysis using the 5′, 3′ and Neo probes. Generation of chimeric mice was performed as described previously (Mishina & Sakimura, 2007). Briefly, targeted clones were microinjected into eight‐cell‐stage embryos of a CD‐1 mouse strain. Resulting chimeric embryos were developed to the blastocyst stage by incubation for more than 24 h and then the blastocysts were transferred into the uteri of pseudopregnant surrogate CD‐1 mice that were anaesthetized by inhalation of vaporized isoflurane (2−3%) using NARCOBIT‐E(II) (KN‐1071, Natsume Seisakusho, Japan). Germline chimeras were crossed with C57BL/6N female mice to establish the *Kcnh7*‐flox mouse line. To generate *Kcnh7* KO mice by Cre‐mediated recombination, heterozygous *Kcnh7*‐flox mice were crossed with Actb‐iCre mice (Zhou *et al*. 2018), which ubiquitously express Cre recombinase. Genotyping of KO mice was performed by genomic PCR with the following specific primers: (forward), 5′‐TGTAGAGACTCCGTGGATC‐3′; (reverse), 5′‐GAAATCTCAACTAAACTTC‐3′.

### HEK293 cells

HEK293 cells were purchased from JCRB Cell Bank (JCRB Cell Bank Cat No. JCRB9068, RRID: CVCL_0045, Osaka, Japan), who stated authentication. The cells were maintained in Dulbecco's modified Eagle's medium (DMEM; Nacalai Tesque, Kyoto, Japan) supplemented with 10% fetal bovine serum, 100 U ml^−1^ of penicillin, and 100 μg ml^−1^ of streptomycin, under humidified air at 37°C in 5% CO_2_. Cells were grown in glass‐bottomed dishes (μ‐Dish 35 mm low; ibidi, NIPPON Generics, Tokyo, Japan).

### Slice preparation

Coronal brain slices (250 μm) including the IO were prepared from C57BL6/J and *Kcnh7* KO littermates at P14−20. Mice were placed in the chamber and then the CO_2_ level was increased. After losing consciousness, mice were decapitated. A block of the brain containing the medulla oblongata was removed and put into ice‐cold cutting solution composed of (in mm) 210 sucrose, 10 glucose, 26 NaHCO_3_, 5 KCl, 1.2 KH_2_PO_4_, 1.3 MgSO_4_, and 2.4 CaCl_2_, bubbled with mixed gases (95% O_2_ and 5% CO_2_). The block of medulla was embedded in 1.5% agarose, and coronal slices were prepared using a vibrating blade microtome (VT‐1200S, Leica, Wetzlar, Germany). Slices were incubated for more than 1 h at room temperature in normal external solution composed of (in mm): 125 NaCl, 2.5 KCl, 2 CaCl_2_, 1 MgSO_4_, 1.25 NaH_2_PO_4_, 26 NaHCO_3_, and 20 glucose, bubbled with 95% O_2_ and 5% CO_2_.

### Expression vectors

Wild‐type mouse *Kcnh2* (NM_013569.2 (Erg1a), NM_001294162.1 (Erg1b)), *Kcnh7* (NM_133207.2), *Hcn1* (NM_010408.3) and *Pex5l* (XM_006535506.3) expression vectors were purchased from VectorBuilder (Santa Clara, CA, USA). The CMV promoter was used, and the channel coding sequence was followed by IRES and EGFP (pCMV‐*Kcnh7* and pCMV‐*Kcnh2*) or mCherry (pCMV‐*Hcn1* and pCMV‐*Pex5l*).

### Heterologous expression in HEK293 cells

HEK293 cells were grown in glass‐bottomed dishes for 24 h following transfection with expression vectors using Lipofectamine LTX (Thermo Fisher Scientific, Waltham, MA, USA) according to the manufacturer's instructions. After 24−72 h, the glass‐bottomed dishes were placed on the microscope stage and used for whole‐cell recording.

### Electrophysiology

**Figure 1:**
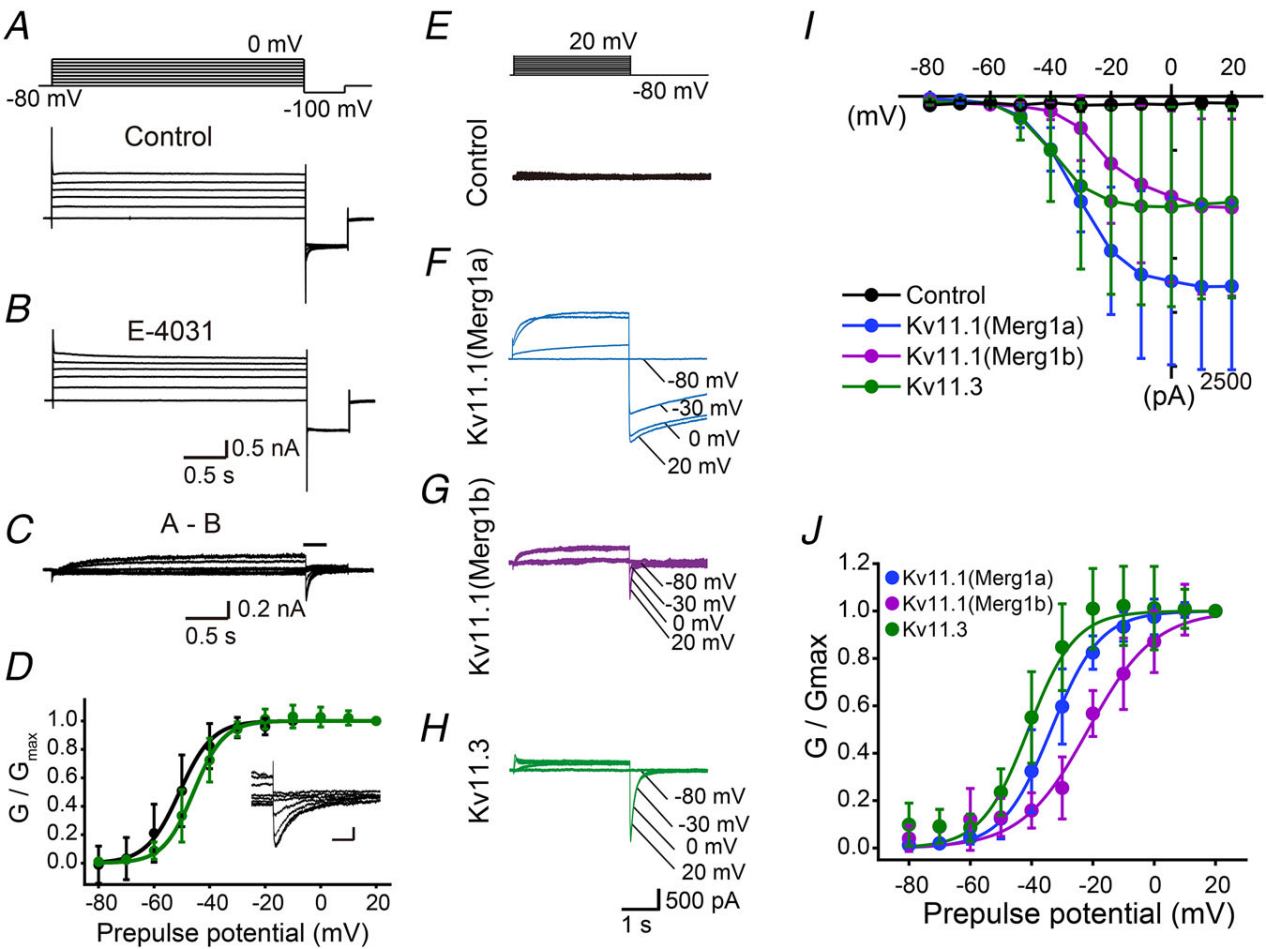
IO neurons exhibit Kv11 conductance *A*, top panel shows waveform of input voltage steps to evoke Kv11 current. Bottom panel shows representative current traces recorded from an IO neuron in control high K^+^ external solution. *B*, representative current traces in the presence of E‐4031 (10 μm). *C*, current traces calculated by subtracting traces in the presence of E‐4031 (*B*) from those in the control solution (*A*, bottom). *D*, relative conductance‐voltage plot of tail currents of IO neurons (*n* = 6, animals = 5, black) and the Kv11.3 current recorded at 32°C (*n* = 5, green). Lines are a theoretical fit using the Boltzmann equation. Inset: elongated traces at a point indicated by a bar in *C*. Scale bars, 0.1 nA, 50 ms. Data are represented as the meas ± SD. *E*, upper panel shows input voltage steps to evoke Kv11 currents in HEK293 cells. Lower traces are representative current traces recorded from a control HEK293 cell in response to the voltage steps. *F*–*H*, representative current traces recorded from a HEK293 cell transfected with Kv11.1(Merg1a) (*F*), Kv11.1(Merg1b) (*G*), or Kv11.3 (*H*). *I*, the *I‐V* plot of Kv11 tail currents in response to hyperpolarizing voltage steps to −80 mV in Kv11.1(Merg1a) (*n* = 6), Kv11.1(Merg1b) (*n* = 7), or Kv11.3 (*n* = 9) transfected cells. *J*, relative conductance‐voltage plots. Individual data points are calculated from the data in *I*. Blue, purple and green lines are theoretical fits using the Boltzmann equation for Kv11.1(Merg1a), Kv11.1(Merg1b), or Kv11.3, respectively. Data are represented as the means ± SD.

**Figure 2:**
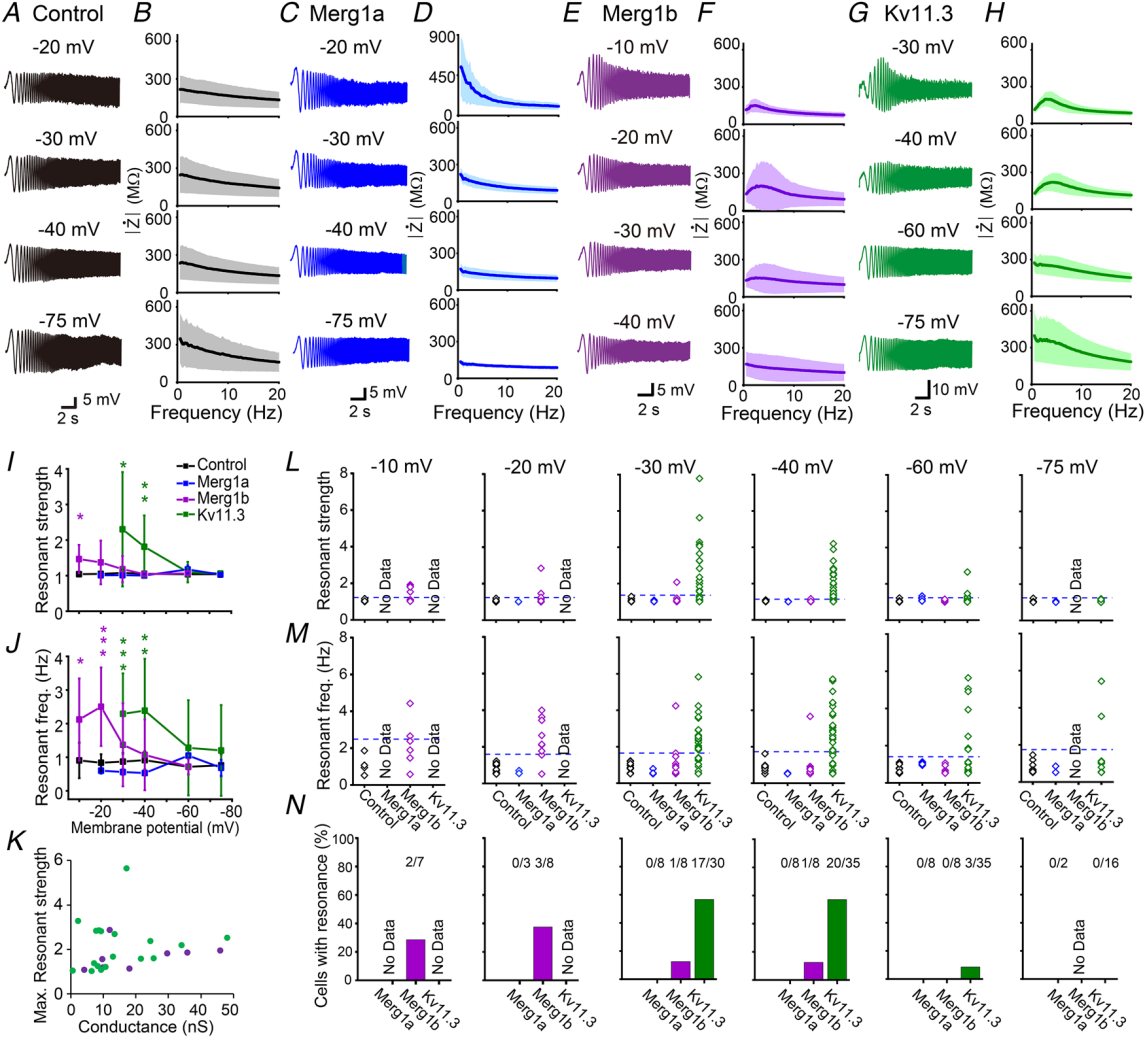
Heterologous expression of Kv11.1(Merg1b) or Kv11.3 induces resonance in HEK293 cells *A*, *C*, *E*, *G*, representative voltage waves of a control HEK293 cell (*A*) and those transfected with Kv11.1(Merg1a) (*C*), Kv11.1(Merg1b) (*E*), or Kv11.3 (*G*) in response to chirp currents (±50 pA) at individual membrane potentials. *B*, *D*, *F*, *H*, average *Z‐F* profiles of control HEK293 cells (*B*, *n* = 6−11 cells) and those transfected with Kv11.1(Merg1a) (*D*, *n* = 2−8), Kv11.1(Merg1b) (*F*, *n* = 7−8), or Kv11.3 (*H*, *n* = 16−35). Shaded areas indicate the mean ± 95% confidence interval. *I* and *J*, membrane potential dependency of the resonant strength (*I*) and resonant frequency (*J*) in control (*n* = 6−11, black) and Kv11.1(Merg1a)‐ (*n* = 2−8, blue), Kv11.1(Merg1b)‐ (*n = *7−8, purple), and Kv11.3‐expressing (*n* = 16−35, green) HEK293 cells. Significance of Kv11‐expressing cells was compared with that of control cells at each membrane potential. ^***^ *P < *0.001, ^**^ *P* < 0.01, ^*^ *P* < 0.05 (one‐way ANOVA, *post hoc*; Holm–Šidák test). Data are presented as the means ± SD. *K*, relationship between conductance and maximum resonant strength in individual Kv11.1(Merg1b)‐ (purple) or Kv11.3 (green)‐expressing cells, regardless of recorded membrane potentials. Kv11.3 conductance was calculated by the linear regression of tail‐current amplitudes evoked by depolarizations to −40, −30 and −20 mV. Kv11.1(Merg1b) conductance was calculated from depolarizations to −20, −10 and 0 mV. *L* and *M*, individual data plots of the resonant strength (*L*) and resonant frequency (*M*) presented in *I* and *J* in control HEK293 cells and those transfected with Kv11.1(Merg1a), Kv11.1(Merg1b), and Kv11.3. Dashed lines represent thresholds calculated from data of control HEK293 cells (average + 3 × SD) at individual membrane potentials. Because of the generation of oscillation and/or severe membrane potential instability, resonance was only able to be recorded up to −10, −20 and −30 mV in Kv11.1(Merg1b)‐, Kv11.1(Merg1a)‐ and Kv11.3‐expressing cells, respectively. *N*, the percentage of HEK293 cells with resonance at individual membrane potentials.

**Figure 3:**
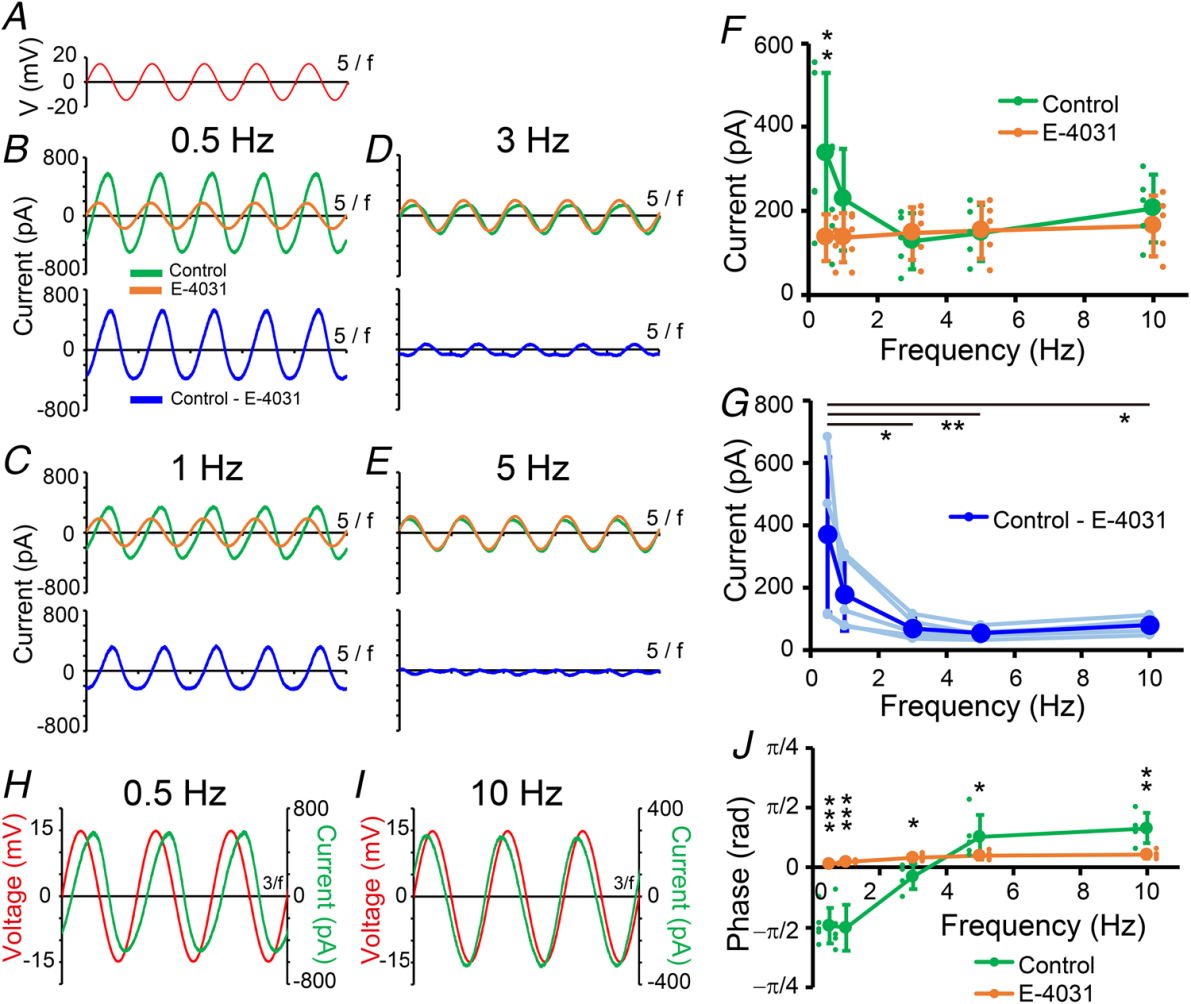
Kv11.3 current declines with input voltage frequency and mediates the lagged phase shift of the membrane current *A*, the waveform of the sinusoidal input voltage. *B*–*E*, upper traces show representative current traces recorded in control (green) and E‐4031‐containing (orange) solutions in response to the sinusoidal voltage clamp (±15 mV) with a frequency of 0.5 Hz (*B*), 1 Hz (*C*), 3 Hz (*D*), and 5 Hz (*E*) at −30 mV in Kv11.3‐expressing HEK293 cells. To avoid transient responses, waves from the fourth to the eighth cycles of input voltage were analysed. Lower traces are E‐4031‐sensitive currents (blue) calculated by subtracting the currents recorded in the presence of E‐4031 from those recorded in the control solution. *F*, amplitudes of membrane currents of control (green) (*n* = 5) and E‐4031‐containing (orange) solutions (*n* = 5) plotted against holding potential frequency. The half‐magnitude from the bottom to the top of the sinusoidal membrane current represents amplitude. ^**^ *P* < 0.01 between control and E‐4031 (*P* = 0.023, two‐way ANOVA, *post hoc*, Holm–Šidák test). *G*, frequency dependence of amplitudes of E‐4031‐sensitive currents (blue, *n* = 5) (*P* = 0.003, one‐way ANOVA, *post hoc*, Holm–Šidák test). *H* and *I*, phase between total current (green) and holding voltage (red) with 0.5 Hz (*H*) and 10 Hz (*I*). These traces were recorded from the same cell shown in *B*–*E*. *J*, phase of the membrane current relative to the holding potential frequency (control, *n* = 5; E‐4031, *n* = 5). The phase was estimated by cross‐correlation analysis. The phases of the membrane currents changed from lag to lead in the control solution, but they were always lead in the presence of E‐4031. ^***^ *P < *0.001, ^**^ *P* < 0.01, ^*^ *P* < 0.05 between control and E‐4031 (*P* < 0.001, two‐way ANOVA, *post hoc*, Holm–Šidák test). Data are presented as the means ± SD.

**Figure 4:**
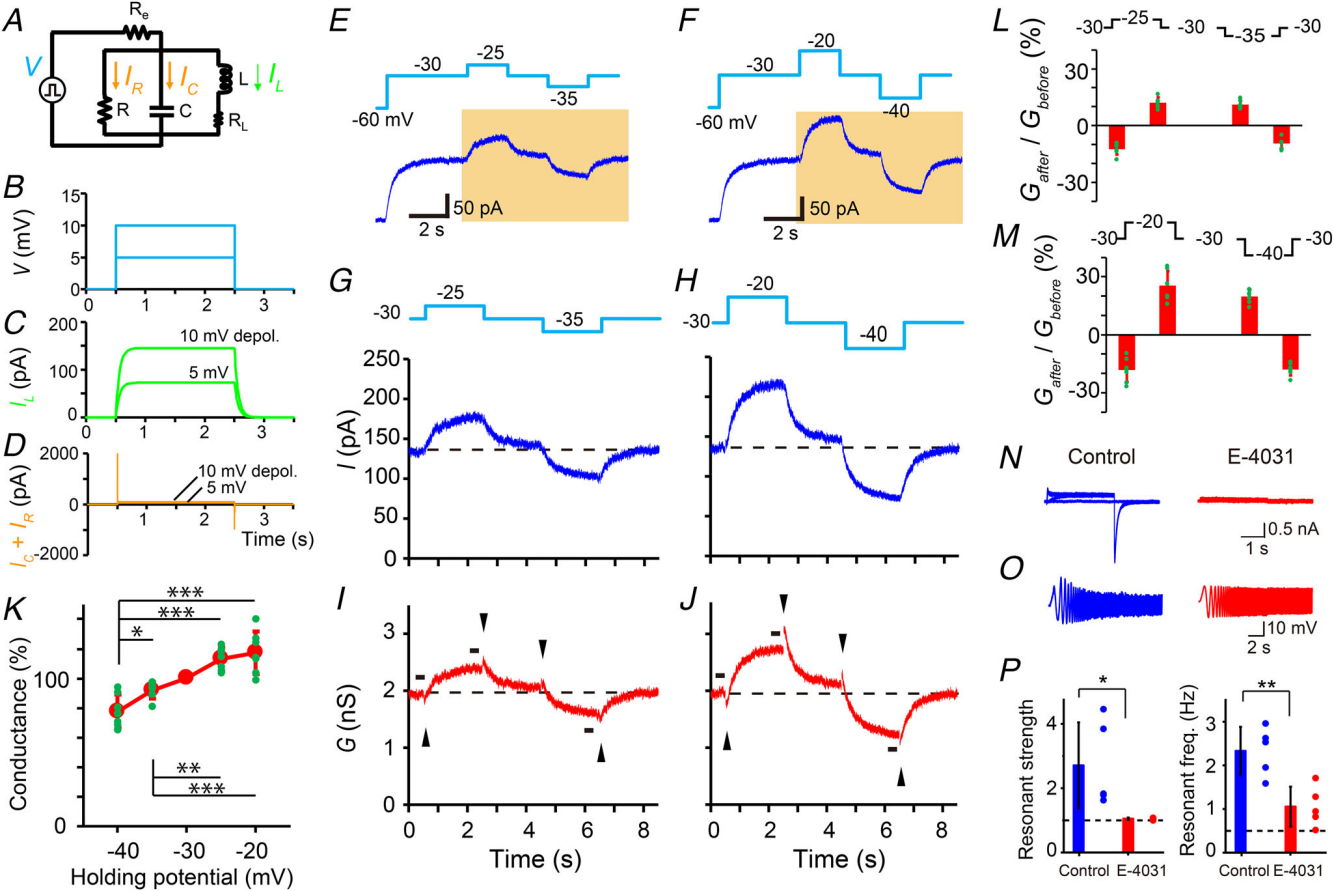
The Kv11.3 current shows inductor‐like responses to the voltage steps *A*, the RLC circuit with a leaky inductor (L and R_L_) and resistance of the recording electrode (R_e_). *B*, waveform of input voltage steps with amplitudes of 5 and 10 mV. *C*, simulated currents flowing through the leaky inductor (*I*_L_) in response to depolarizing voltage steps. Currents were simulated by LTspice XVII. *D*, simulated currents flowing through the resistor and capacitor (*I*_C_ + *I*_R_). *E* and *F*, upper traces show voltage protocols applied to Kv11.3‐expressing cells. Lower traces are representative Kv11.3 currents. Kv11.3 currents were calculated by subtracting currents recorded in the presence of E‐4031 from those in the control external solution. *G* and *H*, the expanded traces in response to 5 mV (*G*) or 10 mV (*H*) voltage steps at a shaded period in *E* or *F*. *I* and *J*, Kv11.3 conductance calculated from data in *G* or *H*. Conductance was calculated by dividing the current amplitude by the driving voltage (holding potential–theoretically calculated K^+^ equilibrium potential (−99 mV)). Black arrowheads, transient opposing conductance changes. Black bars, time windows for steady‐state conductance measurements in *K*. *K*, steady‐state Kv11.3 conductance (black bars in *I* and *J*) relative to those at −30 mV (*n* = 7). Green spots represent individual data. ^***^ *P < *0.001, ^**^ *P* < 0.01, ^*^ *P* < 0.05 (*P* < 0.001, two‐way ANOVA, *post hoc*, Holm–Šidák test). *L* and *M*, magnitudes of opposing conductance changes (arrowheads in *I* and *J*) relative to steady‐state conductance before onsets or offsets of 5 mV (*L*) or 10 mV (*M*) depolarizing (left two bars) or hyperpolarizing (right two bars) voltage steps from −30 mV (*n* = 7). Data are represented as the means ± SD. *N*, representative waves recorded in the absence (left) or presence (right) of E‐4031 (10 μm) in a Kv11.3‐expressing HEK293 cell in the voltage‐clamp mode. Similar results were obtained from eight cells. *O*, representative voltage waves of a Kv11.3‐expressing HEK293 cell in response to chirp currents (50 pA) at membrane potentials of −30 mV in the absence (left) or presence (right) of E‐4031. Waves were recorded in the current‐clamp mode. *P*, effects of E‐4031 on the resonant strength (left) and resonant frequency (right) at −30 mV (*n* = 5). Resonance was totally blocked by E‐4031. Data are presented as the means ± SD. ^*^ *P* < 0.05, ^**^ *P* < 0.01 (*t*‐tests).

**Figure 5:**
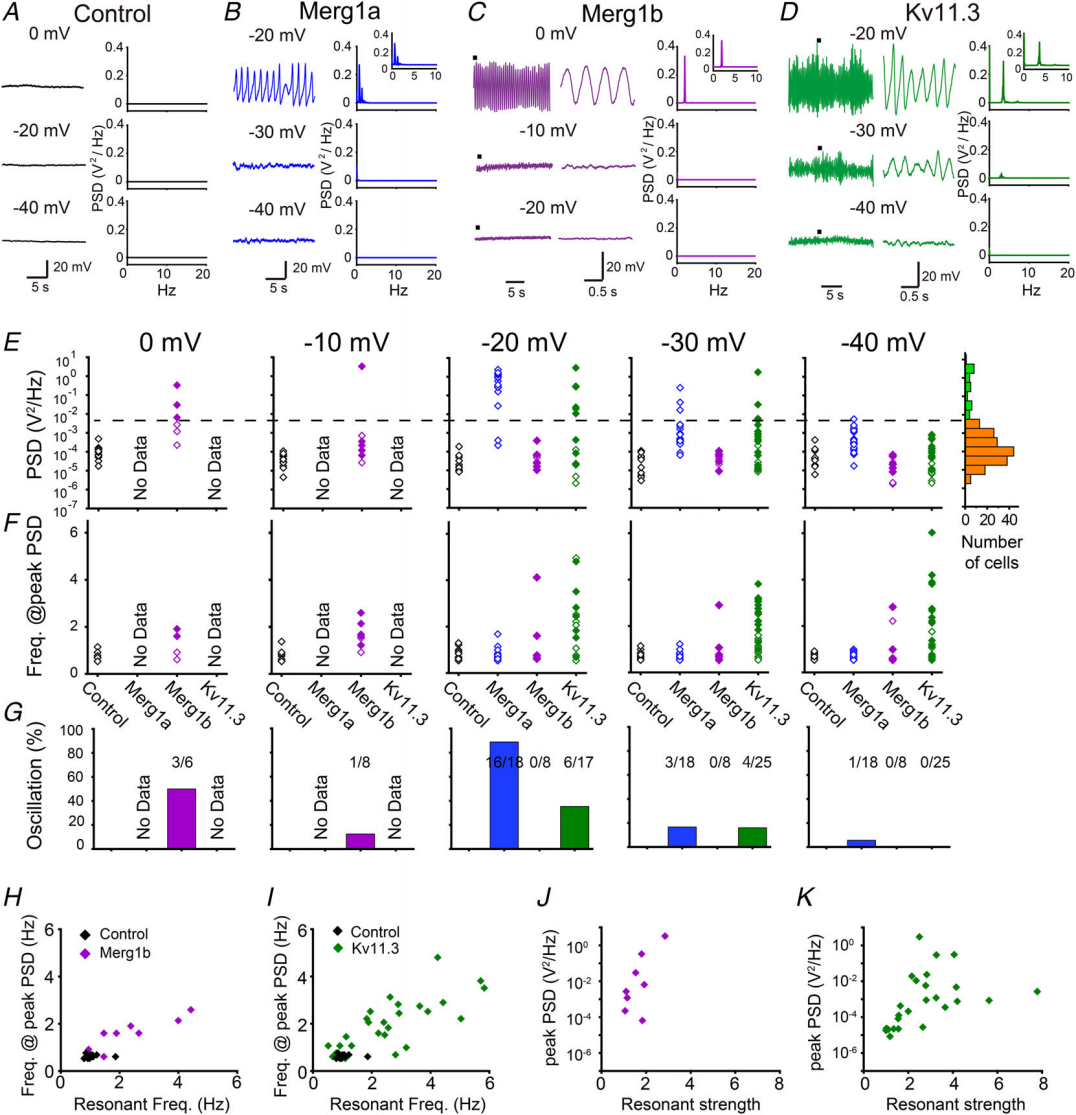
Heterologous expression of Kv11 channels generates membrane potential oscillation *A* and *B*, left traces are representative traces in a control HEK293 cell (*A*) and cells transfected with Kv11.1(Merg1a) (*B*) at individual membrane potentials. Right panels are PSDs calculated from the left traces by FFT. Inset: the PSD from 0−10 Hz is expanded. *C* and *D*, left traces are representative traces in cells transfected with Kv11.1(Merg1b) (*C*), or Kv11.3 (*D*) at individual membrane potentials. Middle panels show elongated traces at points indicated by bars in individual left traces. Right panels are PSDs calculated from the left traces by FFT. *E*, magnitude of peak PSDs at 0, −10, −20, −30 and −40 mV in control HEK293 cells (*n* = 7−11, black) and cells transfected with Kv11.1(Merg1a) (*n* = 18, blue), Kv11.1(Merg1b) (*n* = 6−8, purple) and Kv11.3 (*n* = 17−25, green). Filled symbols represent cells with resonance. Right panel shows distribution of all peak PSDs. Data were classified into two groups using *k*‐means clustering (green and orange). The dashed line represents the threshold between two groups (10^−2.33^ V^2^ Hz^−1^). *F*, individual data of the peak PSD frequency. *G*, the percentage of HEK293 cells whose peak PSDs were larger than 10^−2.33^ V^2^ Hz^−1^. *H* and *I*, the relationship between resonant frequency and peak PSD frequency in control HEK293 cells and those transfected with Kv11.1(Merg1b) (*H*) and Kv11.3 (*I*). Only cells with recordings of both resonance and oscillation are plotted. *J* and *K*, the relationship between resonant strength and peak PSD in HEK293 cells transfected with Kv11.1(Merg1b) (*J*) and Kv11.3 (*K*).

**Figure 6:**
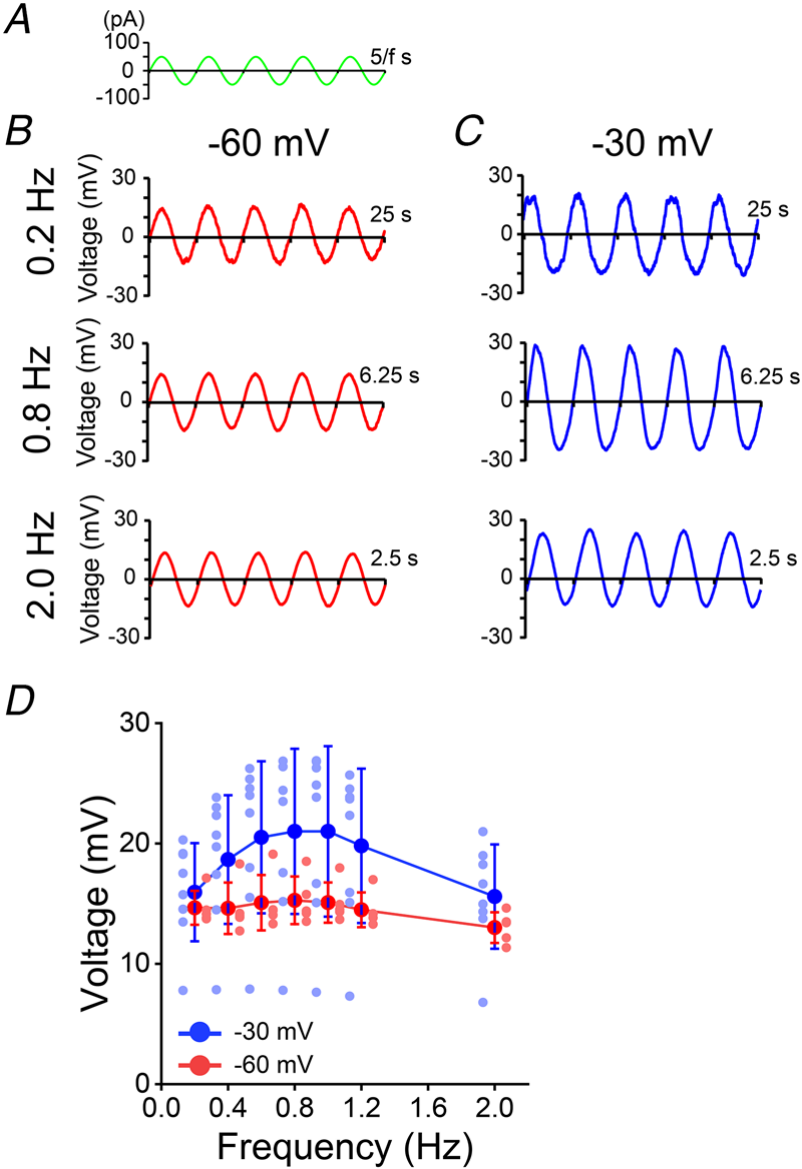
Kv11.1(Merg1a)‐expressing cells show resonance *A*, the waveform of the sinusoidal input current. *B* and *C*, representative currents recorded in a Kv11.1(Merg1a)‐expressing cell in response to the sinusoidal input currents (±50 pA) with a frequency of 0.2 Hz (top), 0.8 Hz (middle) and 2.0 Hz (bottom) at −60 mV (*B*) and –30 mV (*C*). To avoid transient responses, voltage waves from the fourth to the eighth cycles of the input current were analysed. *D*, magnitudes of sinusoidal membrane potentials at −60 mV (*n* = 5, red) and −30 mV (*n* = 8, blue) plotted against frequencies of input currents. Data are presented as the means ± SD.

**Figure 7:**
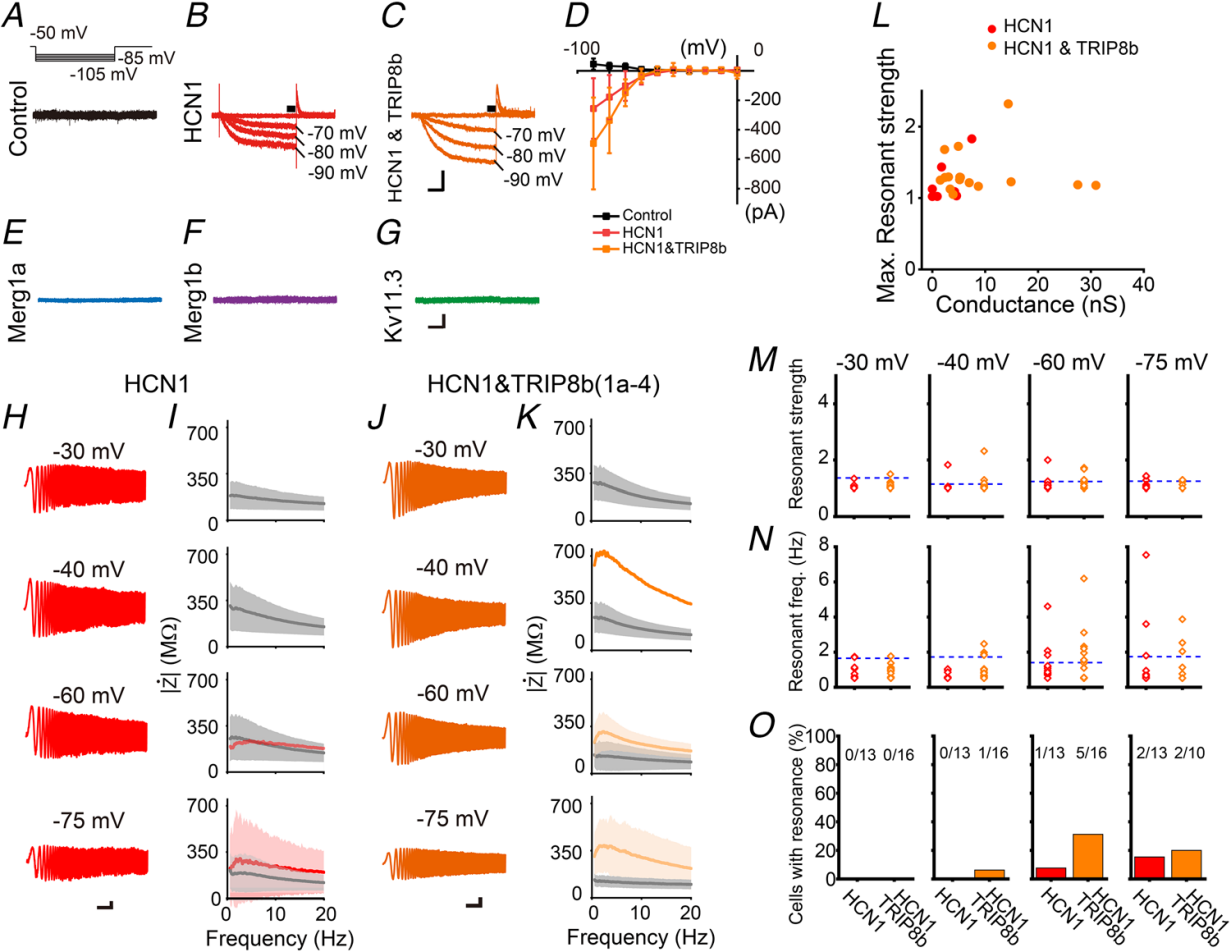
HCN1‐expressing cells exhibit resonance at hyperpolarized potentials *A*, upper traces show hyperpolarizing voltage steps to evoke HCN currents. Lower traces are representative current traces in a control HEK293 cell. *B*, *C*, *E*–*G*, representative current traces recorded in an HCN1‐ (*B*), HCN1 + TRIP8b‐ (*C*), Kv11.1(Merg1a)‐ (*E*), Kv11.1(Merg1b)‐ (*F*), or Kv11,3‐expressing (*G*) HEK293 cell. Scale bars, 200 pA, 0.2 s. *D*, *I*‐*V* plot of HCN currents in response to hyperpolarizing voltage steps in control (*n* = 7), HCN1 (*n* = 11) and HCN1 + TRIP8b (*n* = 10) transfected cells. Data are represented as the means ± SD. *H*, representative voltage waves of an HCN1‐expressing HEK293 cell in response to chirp currents (±50 pA) at individual membrane potentials. Scale bars, 10 mV, 2 s. *I*, average *Z‐F* profiles of HCN1 cells (*n* = 13 cells). Shaded area indicates the mean ± 95% confidence interval. Red and grey lines represent data from cells with (*n* = 1−2) or without (*n* = 11−13) resonance, respectively. *J*, similar to *H*, but with voltage traces recorded from a HEK293 cell transfected with HCN1 and TRIP8b. *K*, averaged *Z‐F* profiles of HEK293 cells transfected with HCN1 and TRIP8b (*n* = 10−16). Orange and grey lines represent data from cells with (*n* = 1−5) or without (*n* = 8−16) resonance, respectively. *L*, relationship between HCN1 conductance and the maximum resonant strength in HCN1‐ (red) and HCN1 + TRIP8b‐ (orange) expressing cells regardless of recorded membrane potential. *M* and *N*, resonant strength (*M*) and resonant frequency (*N*) at −30, −40, −60 and −75 mV in HEK293 cells transfected with HCN1 (*n* = 13) and HCN1 + TRIP8b (*n* = 10−16). Dashed lines represent thresholds calculated from data of control HEK293 cells (average + 3 × SD, Fig. 2 *L* and *M*) at individual membrane potentials. *O*, the percentage of HEK293 cells with resonance at individual membrane potentials.

Whole‐cell recordings were made from neurons in the principal or dorsal accessory olive (see Figs 1 *A*–*D* and 10) or HEK293 cells (see Figs 1 *D*–*J*, 2‐7) using an upright microscope (BX51WI; Olympus, Tokyo, Japan) equipped with an IR‐CCD camera system (IR‐1000, DAGE‐MTI, Michigan City, IN, USA). For recording from IO neurons, the temperature was kept at 32°C using an inline heater (TC‐324B, Warner, Holliston, MA, USA). Recording from HEK293 cells was performed at room temperature except in Fig. 1 *D*.

For voltage‐dependent K^+^ current recordings (see Figs 1, 4 *N*, 10 *A*–*C*), the low K^+^ intracellular solution was composed of (in mm) 55 potassium methanesulfonate, 5 KCl, 73 *N*‐methyl‐d‐glucamine, 5 NaCl, 0.5 EGTA, 30 HEPES, 4 MgCl_2_, 4 ATP disodium salt (2Na‐ATP) and 0.4 GTP disodium salt (2Na‐GTP; pH 7.4, adjusted with KOH). Voltage‐dependent K^+^ currents of HEK293 cells were recorded in the high K^+^ and Ca^2+^‐free external solution that was composed of (in mm) 117.5 NaCl, 10 KCl, 3 MgSO_4_, 1.25 NaH_2_PO_4_, 26 NaHCO_3_, and 20 glucose, bubbled with 95% O_2_ and 5% CO_2_. For recording of Kv11 currents from IO neurons, 0.5 μm tetrodotoxin (TTX), 10 μm NBQX, 5 μm (R)‐CPP, and 10 μm bicuculline were also supplemented. These high K^+^ extracellular and low K^+^ intracellular solutions enable K^+^ currents to be recorded as large, distinct inward currents (Sturm *et al*. 2005). The theoretically calculated equilibrium potential of K^+^ was −46.0 mV at room temperature and −47.1 mV at 32°C. The pipette access resistance was approximately 3−4 MΩ. Relative conductance plots were fitted using the following Boltzmann equation:

$$\frac{G}{G_{\max}}=\frac{1}{1+exp\left(\left(V_{\mathrm{half}}-V_{m}\right)/k\right)}.$$

The intracellular solution used for recording resonance and oscillation (see Figs 2, 3, 4 *A*–*M*, *O*, *P*; 5−7, 10 *D*–*L*) was composed of (in mm) 115 potassium methanesulfonate, 5 KCl, 5 NaCl, 0.5 EGTA, 30 HEPES, 4 MgCl_2_, 4 2Na‐ATP and 0.4 2Na‐GTP (pH 7.3, adjusted with KOH). For experiments using HEK293 cells, resonance and oscillation were recorded in normal external solution with 0.5 μm TTX. For recording from IO neurons, Ca^2+^‐free external solution was used to suppress Cav3.1‐dependent amplifying conductance (Matsumoto‐Makidono *et al*. 2016) (see Fig. 10 *D*–*J*). The composition was (in mm): 125 NaCl, 2.5 KCl, 3 MgSO_4_, 1.25 NaH_2_PO_4_, 26 NaHCO_3_, 20 glucose, 0.0005 TTX, 0.01 NBQX, 0.005 (R)‐CPP and 0.01 bicuculline, bubbled with 95% O_2_ and 5% CO_2_. The pipette access resistance was approximately 3−4 MΩ.

Ionic currents were recorded with an EPC‐10 (HEKA Elektronik, Lambrecht/Pfalz, Germany). The signals were filtered at 3 kHz and digitized at 20 kHz in experiments for measuring Kv11 currents, or at 1 kHz in experiments for resonance. On‐line data acquisition and off‐line data analysis were performed using PATCHMASTER software (v2×90.2, HEKA). The liquid junction potentials were not corrected.

If the electrode potential polarization was over ±5 mV from the initial value at the end of the recording, the recording was omitted from the analysis. Furthermore, recordings with high series resistances (over 10 MΩ) were also omitted. For the impedance amplitude profile (ZAP) protocol, recordings with large fluctuations or positive or negative voltage shifts of the baseline potential were omitted. In the analysis of IO neurons, traces with Cav3.1‐dependent amplifying conductance were also omitted.

### Impedance measurement

Impedance was measured using the ZAP protocol in the non‐oscillating condition as described previously (Puil *et al*. 1986; Hutcheon & Yarom, 2000). Sinusoidal currents whose amplitudes were constant (50 pA) but frequencies were linearly changed in a range from 0−40 Hz in 40 s (a chirp current) were applied from a recording electrode under the current‐clamp mode. Impedance was calculated by dividing the fast Fourier transform (FFT) of the voltage response by the FFT of the chirp current.

$$\dot{Z}\left(f\right)=\frac{\mathrm{FFT}\;\mathrm{of}\;\mathrm{voltage}\;\mathrm{response}}{\mathrm{FFT}\;\mathrm{of}\;\mathrm{chirp}\;\mathrm{current}},$$

where $\dot{Z}\left(f\right)$ and f denote the complex impedance and frequency of the input current, respectively. The magnitude of impedance was calculated as follows:

Z˙f=Z˙fRe2+Z˙fIm2,

Where Z˙(f)Re and Z˙(f)Im are the real and imaginary components of impedance, respectively. For calculating the impedance of HEK293 cells, another chirp current with a phase shift of 180° was also applied, and the magnitude of impedance was calculated by averaging two impedances recorded by phases of 0° and 180°. For analysis of IO neurons, only data of phases of 0° were used (Matsumoto‐Makidono *et al*. 2016). The frequency (higher than 0.5 Hz) at which impedance reached the maximum value was termed the resonant frequency. The resonant strength was calculated as the ratio of the maximum impedance amplitude at the resonant frequency relative to the impedance amplitude at 0.5 Hz in HEK293 cells. These calculations were performed with Excel (Microsoft, WA, USA), Origin 2018 (OriginLab, Northampton, MA, USA), and Igor Pro 6.3.7 (WaveMetrics, Portland, OR, USA). When resonance does not occur or is blocked, impedance monotonically declines with input frequency. In such cases, the resonant frequency is equal or near to 0.5 Hz and the resonant strength is equal or near to 1. The resonant strength for IO neurons was calculated in a similar way, but relative to impedance at 1 Hz (Matsumoto‐Makidono *et al*. 2016).

### Fluorescent *in situ* hybridization

We employed non‐isotopic *in situ* hybridization with fluorescein‐ and digoxigenin (DIG)‐labelled cRNA probes for Kv11.3 (nucleotides 2840−3580 bp according to the Allen Brain Atlas; GenBank accession number, NM_133207.2) and VGluT2 (934−2060 bp; BC038375.1) mRNAs. cRNA probes were synthesized by *in vitro* transcription using the Bluescript II plasmid vector encoding the above cDNAs, as described previously (Yamasaki *et al*. 2010). Under deep pentobarbital anaesthesia (100 μg g^−1^ body weight, i.p.), C57BL/6J mice were decapitated. Brains were freshly obtained and immediately frozen in powdered dry ice. Frozen sections (20 μm) were cut on a cryostat (CM1900; Leica Microsystems, Wetzlar, Germany) and mounted on silane‐coated glass slides. Digoxigenin (DIG)‐ or fluorescein‐labelled cRNA probes were prepared for simultaneous detection of multiple mRNAs by fluorescent *in situ* hybridization (Yamasaki *et al*. 2010). In brief, fresh frozen sections were hybridized with a mixture of DIG‐ or fluorescein‐labelled cRNA probes diluted to 1:500 with the hybridization buffer. After stringent posthybridization washing, DIG and fluorescein were detected using the two‐step method as follows: the first detection with peroxidase‐conjugated anti‐fluorescein antibody (1:1000, 1.5 h, Roche Diagnostics, Risch‐Rotkreuz, Switzerland) and the FITC‐TSA plus amplification kit (PerkinElmer, Waltham, MA, USA), and the second detection with peroxidase‐conjugated anti‐DIG antibody (1:1000, 1.5 h, Roche Diagnostics) and the Cy3‐TSA plus amplification kit (PerkinElmer). Residual activities of the peroxidase introduced in the first detection were inactivated by incubating the sections with 1.0% H_2_O_2_ for 30 min. After extensive washing and blocking with 10% donkey serum, sections were incubated with 1 μg ml^−1^ of anti‐NeuN antibody (MAB377, Merck, Darmstadt, Germany) for 1 h, and Alexa 647‐conjugated anti‐mouse IgG (Invitrogen) for 2 h. Images were taken with a laser scanning microscope (FV1200, Olympus, Tokyo, Japan) equipped with 473, 559 and 635 nm diode laser lines and UPlanSApo (20×/0.75) and PlanApoN (60×/1.40 oil immersion) objective lenses (Olympus). All images represent single optical sections.

### Antibodies

We used the following primary antibodies: rabbit anti‐Kv11.3, guinea‐pig anti‐HCN1 (Frontier Institute, Cat. No. HCN1‐GP‐Af540, RRID: AB_2650995, Hokkaido, Japan), goat anti‐microtubule‐associated protein 2 (MAP2; Cat. No. MAP2‐Go‐Af860, RRID: AB_2571793), and goat anti‐green fluorescent protein (GFP; Cat. No. GFP‐Go‐Af1480, RRID: AB_2571574) (Matsumoto‐Makidono *et al*. 2016). Of these, we produced the affinity‐purified Kv11.3 antibody in the present study, which was raised against amino acid residues 1171−1195 of mouse Kv11.3 (NP_573470.2). The specificity of the Kv11.3 antibody was confirmed by blank immunohistochemical labelling in *Kcnh7* KO brains at light microscopic levels (Fig. 9 *D* and *E*).

### Viral vector preparation and injection

For retrograde labelling of IO neurons, lentivirus expressing GFP was prepared as described previously (Matsumoto‐Makidono *et al*. 2016). P15 C57BL/6J mouse pups were anaesthetized by inhalation of vaporized isoflurane (4% induction, 2% maintenance). The lentivirus solution (0.3 μl/site) was injected by air pressure into the vermis of cerebellar lobules 6−7. After 14 days of survival, mice were processed for immunohistochemistry.

### Immunofluorescence

Under deep pentobarbital anaesthesia (100 μg g^−1^ body weight, i.p.), P26−29 C57BL/6J and *Kcnh7* KO littermates were transcardially fixed with 4% paraformaldehyde in 0.1 m PB (pH 7.2). After 4 h post‐fixation in the same fixative, coronal sections (50 μm) were made with a microslicer (VT1000S, Leica Microsystems). The following incubations were performed at room temperature (25°C). Sections were incubated with 10% normal donkey serum for 20 min, a mixture of primary antibodies (1 μg ml^−1^ each) overnight, and a mixture of Alexa Fluor 488‐, Alexa Fluor 647‐ (Invitrogen), and Cy3‐ (Jackson Immunoresearch, West Grove, PA, USA) conjugated species‐specific secondary antibodies for 2 h at a dilution of 1:200. Images were taken with a laser scanning microscope (FV1200, Olympus) using a PlanApoN (60×/1.40 oil immersion) objective lens (Olympus). To avoid cross‐talk between multiple fluorophores, fluorescent signals were acquired sequentially using individual excitation laser lines with a 3× digital zoom and an appropriate pinhole (120 μm). Images were digitized at 12‐bit resolution into an array of 800 × 800 pixels (pixel size, 0.1 μm). Single optical section imaging was used for triple immunostaining of MAP2, HCN1 and Kv11.3. For immunofluorescence in virally GFP‐labelled single neurons, a maximum‐intensity *z*‐projection image was produced from four optical sections taken at intervals of 0.75 μm.

### Drugs

E‐4031 (Cat. No. 1808, CAS 113559‐13‐0), ZD7288 (Cat. No. 1000, CAS 133059‐99‐1), NBQX (Cat. No. 0373, CAS 118876‐58‐7), and (R)‐CPP (Cat. No. 0247, CAS 126453‐07‐4) were purchased from Tocris Bioscience (Bristol, UK). TTX (Cat. No. 32775‐51, CAS 4368‐28‐9) was obtained from Nacalai Tesque.

### Statistics

Throughout the text, data are represented as the means ± SD. Data in the figures are also represented as the means ± SD, except for the shaded areas in the impedance–frequency plot (*Z‐F* plot), which are represented as the means ± 95% confidence interval. ‘*n*’ represents the number of HEK293 cells or IO neurons. Statistical significance was assessed using Student's *t* test or the Mann–Whitney *U* test, depending on whether the data sets passed the normality test and the equal variance test. Statistical comparisons among three or more groups were conducted using a one‐way or two‐way ANOVA. When differences were judged to be significant, data were processed using the Holm–Šidák test as a *post hoc* test. All tests were two‐sided. Data analysis was performed using Excel (Microsoft), Origin 2018 (OriginLab), and Igor Pro 6.3.7 (WaveMetrics). Statistical analyses were conducted using SigmaPlot 12.1 (Systat Software, San Jose, CA, USA) and *P*‐values smaller than 0.001 were described as *P* < 0.001; otherwise, actual values were described in the text. Current responses in the RLC circuit was simulated by LTspice XVII (Analog Devices, Norwood, MA, USA). The *k*‐means clustering was performed using Spider 3.3.6 in Anaconda 3 (Austin, TX, USA). Differences between two samples were considered statistically significant if the *P* value was less than 0.05.

## Results

### IO neurons exhibit Kv11 conductance that is mainly mediated by Kv11.3

We previously reported that the resonance of IO neurons around −60 mV was suppressed by E‐4031 (Matsumoto‐Makidono *et al*. 2016). However, the existence of Kv11 conductance has not been examined in IO neurons. To test this point, whole‐cell patch clamp was conducted with IO neurons in acute slices. Voltage steps of 10 mV were applied from −80 to −10 mV, followed by a negative step to −100 mV (Fig. 1 *A*). The Kv11 current was calculated by subtracting the current trace recorded in the presence of E‐4031 (10 μm) from that recorded in the control solution (Fig. 1 *A*–*C*). Tail currents sensitive to E‐4031 were elicited by negative voltage steps to −100 mV (Fig. 1 *C* and *D*). The relative conductance of the tail currents was plotted against the depolarizing voltage pulses and fitted with a Boltzmann equation. Activation started from around −60 mV, and the averaged half‐conductance potential (*V*_half_) and slope factor (*k*) (Table 2) were similar to those of Kv11 conductance in Purkinje cells (−44.3 to −50.7 mV and 4.9 to 5.6 mV) (Sacco *et al*. 2003; Niculescu *et al*. 2013). These results suggest that IO neurons exhibit Kv11 conductance.

To estimate subtypes of the Kv11 current in IO neurons, we transfected Kv11 channels into HEK293 cells. In the present study, we transfected Kv11.1 or Kv11.3 because of their high expression in the IO nucleus (Shi *et al*. 1997; Saganich *et al*. 2001; Perry & Sanguinetti, 2010). For Kv11.1, we analysed two major long and short splice variants with distinct physiological properties: Merg1a and Merg1b (London *et al*. 1997; Hirdes *et al*. 2005; Niculescu *et al*. 2013). Whole‐cell recording was obtained from GFP‐positive cells or control non‐transfected cells without GFP expression, in the same dish, 1−3 days after transfection. The voltage protocol for the Kv11 current did not evoke detectable currents in control HEK293 cells (Fig. 1 *E*), but evoked clear voltage‐dependent currents in cells expressing Kv11 channels (Fig. 1 *F*–*J*). The *V*_half_ of the Kv11.3 tail currents was the most hyperpolarized, followed in order by those of Kv11.1(Merg1a) and Kv11.1(Merg1b) (Fig. 1 *I* and *J* and Table 1). To compare deactivation processes, the time courses of deactivations were fitted with a single exponential. The decay time constants of deactivation at about 90% activation of Kv11.3 or Kv11.1(Merg1b) were significantly faster than that of Kv11.1(Merg1a) (Table 1, Fig. 1 *F*–*H*). These properties were similar to those previously reported for Merg1a, Merg1b, and Kv11.3 (London *et al*. 1997; Shi *et al*. 1997; Schledermann *et al*. 2001; Wimmers *et al*. 2002; Hirdes *et al*. 2005; Sturm *et al*. 2005; Niculescu *et al*. 2013).

**Table 1:** Half‐conductance potential (*V*_half_), slope factor (*k*), and decay time constant of Kv11 tail currents in HEK293 cells

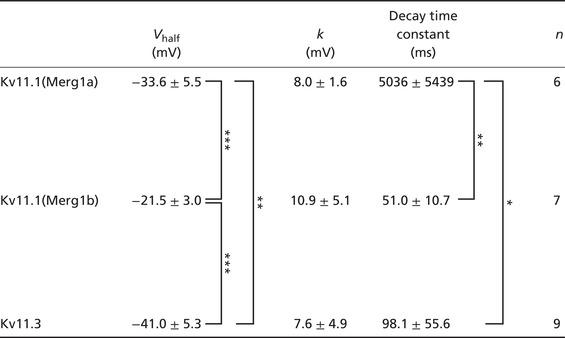

Data are represented as the means ± SD. ^***^ *P* < 0.001, ^**^ *P* < 0.01 (*P* < 0.001, Kv11.1(Merg1a) *vs*. Kv11.1(Merg1b); *P* = 0.008, Kv11.1(Merg1a) *vs*. Kv11.3; *P* < 0.001, Kv11.1(Merg1b) *vs*. Kv11.3; one‐way ANOVA; *post hoc*, Holm–Šidák test). The slope factors were not statistically significant (one‐way ANOVA, *P* = 0.323). Decay time constants at about 90% activations of Kv11.1(Merg1b), Kv11.1(Merg1a), and Kv11.3 were measured from traces recorded at holding potentials of 0, −20, and −30 mV, respectively. ^**^ *P* < 0.01, ^*^ *P* < 0.05 (*P* = 0.009, Kv11.1(Merg1a) *vs*. Kv11.1(Merg1b); *P* = 0.01, Kv11.1(Merg1a) *vs*. Kv11.3; *P* = 0.974, Kv11.1(Merg1b) *vs*. Kv11.3, one‐way ANOVA; *post hoc*, Holm–Šidák test).

The deactivation kinetics of Kv11.1(Merg1a) tail currents were significantly slower (Fig. 1 *F*) than those observed in IO neurons (Fig. 1 *C* and *D*), which suggests that Kv11.1(Merg1a) is not likely to be a major candidate Kv11 channel. Although the deactivation kinetics was similar (Fig. 1 *G*), Kv11.1(Merg1b) was activated at substantially depolarized membrane potentials (Fig. 1 *I* and *J* and Table 1) (Hirdes *et al*. 2005; Niculescu *et al*. 2013) compared with the Kv11 current in IO neurons. In contrast, the deactivation kinetics was fast and membrane potential dependency was most hyperpolarized in Kv11.3 channels (Fig. 1 and Table 1). We further investigated this concept by comparing responses recorded at the same temperature (32°C). The *V*_half_, *k* and decay time constant of Kv11.3 currents recorded in HEK293 cells at 32°C were identical to those of E‐4031‐sensitive currents in IO neurons (Table 2 and Fig. 1 *D*). These data suggest that Kv11.3 is a major Kv11 channel subtype in IO neurons.

**Table 2:** Half‐conductance potential (*V*_half_), slope factor (*k*) and decay time constant of Kv11 tail currents in HEK293 cells and of E‐4031‐sensitive tail currents in IO neurons at 32°C

	*V*_half_	*k*	Decay time constant	*n*	*n*
	(mV)	(mV)	(ms, at −30 mV)	(cells)	(mice)
Kv11.3 current in HEK293 cells	−45.8 ± 4.5	5.5 ± 0.7	52.0 ± 28.1	5	
E‐4031‐sensitive current in IO neurons	−50.8 ± 6.9	6.0 ± 1.7	78.2 ± 46.9	6	5

Data were recorded at 32°C and are represented as the means ± SD. There were no significant differences (*V*_half_, *P* = 0.196; *k*, *P* = 0.547; decay time constant, *P* = 0.304, *t* test).

### Heterologous expression of Kv11.1(Merg1b) or Kv11.3 induces resonance in HEK293 cells

We next examined whether Kv11 channels have the ability to generate resonance. For this purpose, we used the ZAP stimulus (Puil *et al*. 1986; Lampl & Yarom, 1997; Matsumoto‐Makidono *et al*. 2016). In control HEK293 cells, voltage responses gradually declined with the input current frequency at all tested membrane potentials, and there was no clear hump in the impedance–frequency (*Z‐F*) profile, suggesting an absence of resonance (Fig. 2 *A*, *B*, *I*, *J*, *L*–*N*). Likewise, in Kv11.1(Merg1a)‐expressing cells, there was no clear hump in the analysed frequency range from 0.5−40 Hz (Fig. 2 *C*, *D*, *I*, *J*, *L*–*N*). In contrast, in some Kv11.1(Merg1b)‐expressing HEK293 cells, the magnitude of the voltage response was enhanced around 2−4 s (roughly corresponding to 2−4 Hz) at a depolarized potential of −20 mV (Fig. 2 *E*). The *Z‐F* profile exhibited a clear hump (Fig. 2 *F*) at around 2−4 Hz (resonant frequency) (Fig. 2 *J* and *M*). Similar results were also observed in Kv11.3‐expressing cells, but resonance became evident at more hyperpolarized potentials (−40 mV) (Fig. 2 *G*–*J*, *L*, *M*), and the resonant frequency range (2−6 Hz) was higher than that of Kv11.1(Merg1b). In most Kv11.3‐expressing cells, resonance was only able to be recorded up to −30 mV, because membrane potential oscillation was generated and/or the current‐clamp recording often became very unstable at membrane potentials more positive than −30 mV. For similar reasons, resonance was recorded up to −20 and −10 mV in Kv11.1(Merg1a)‐ and Kv11.1(Merg1b) expressing cells, respectively. These data suggest that resonance is induced by the expression of Kv11.1(Merg1b) and Kv11.3 in HEK293 cells.

To estimate the incidence of resonance, we classified resonance as being induced if cells satisfied both of the following criteria at each membrane potential (Fig. 2 *N*): first, the resonant strength was higher than the mean + 3 standard deviations (SD) of control cells. Second, the resonant frequency was higher than the mean + 3 SD of control cells. According to these criteria, approximately 60% of Kv11.3‐expressing cells exhibited resonance at membrane potentials from −40 to −30 mV (Fig. 2 *N*). Resonance became evident at more depolarized potentials (−20 to −10 mV) in Kv11.1(Merg1b)‐expressing cells, but the incidence was less frequent (Fig. 2 *N*). However, even in the Kv11.3‐expressing cells, approximately 40% of cells did not show resonance (Fig. 2 *N*). We assumed that resonant strength might correlate with the magnitude of Kv11 conductance. Although resonant strength likely increased with Kv11.1(Erg1b) or Kv11.3 conductance, the correlation was not significant (Kv11.1(Erg1b), *r* = 0.30, *P* = 0.48 ; Kv11.3, *r* = 0.14, *P* = 0.57, Pearson correlation coefficient) (Fig. 2 *K*). These results suggest that the magnitude of resonant strength is not just affected by the magnitude of Kv11 conductance.

To further confirm whether Kv11 currents are able to show inductor‐like current–voltage responses, Kv11 current responses to sinusoidal voltage inputs were directly recorded under the voltage‐clamp mode. For this analysis, Kv11.3‐expressing cells were examined because they had the highest incidence of resonance and had clear impedance change (Fig. 2). In our previous study, we theoretically analysed the influence of the inductor on current‐voltage responses in the voltage‐clamp mode using RLC circuits (Matsumoto‐Makidono *et al*. 2016; Hashimoto, 2020). The current amplitude flowing through the inductor was predicted to be large at a lower frequency range, but gradually decline with an increase in frequency. Furthermore, the phase of the total current relative to the sinusoidal voltage inputs lagged at the lower frequency range but led at the higher frequency range in the RLC circuit. Similar observations have been also presented in neurons in the current‐clamp recording (Ulrich, 2002; Nolan *et al*. 2007; Narayanan & Johnston, 2008; Zemankovics *et al*. 2010; Dwyer *et al*. 2012; Marcelin *et al*. 2012; Vera *et al*. 2014; Song *et al*. 2016). In contrast, the phase of the total current flowing through the RC circuit, which represents the electrical circuit of the plasma membrane, always leads the voltage inputs, which suggests that the phase lag at the lower frequency range is caused by the inductor. Thus, if Kv11.3 behaves like an inductor, Kv11.3 conductance would be expected to show these properties.

We recorded currents from Kv11.3‐expressing HEK293 cells in response to a sinusoidal voltage clamp with the same amplitude (±15 mV) but different frequencies of 0.5, 1, 3, 5 and 10 Hz at −30 mV (Fig. 3). Current recordings were repeated in the presence or absence of E‐4031, and E‐4031‐sensitive currents (Kv11.3 currents) were calculated by subtracting the traces recorded in the presence of E‐4031 from those in the control solution. The amplitudes of the membrane currents (Fig. 3 *B*–*E*, green) in response to the sinusoidal voltage clamp were smallest around the resonant frequency (Fig. 3 *F*, green) in the control solution. Because input voltage was constant, the smallest current indicated the greatest impedance. In contrast, the amplitudes of the membrane currents monotonically increased in the presence of E‐4031 (Fig. 3 *B*–*E* and *F*, orange), which represented the response in the RC circuit. The isolated E‐4031‐sensitive currents, which represent Kv11.3 currents, exhibited nearly sinusoidal waveforms, and the peak amplitudes declined with increasing input frequencies (Fig. 3 *B*–*E* and *G*, blue). Furthermore, the phase of the membrane current relative to the input voltage shifted from a lag (Fig. 3 *H*) to a lead (Fig. 3 *I*) around the resonant frequency (Fig. 3 *J*, green) in the control solution. They were always lead in the presence of E‐4031 (Fig. 3 *J*, orange), suggesting the loss of the inductor‐like element. These results support the idea that E‐4031‐sensitive Kv11.3 conductance plays an inductor‐like role.

### The Kv11.3 current shows inductor‐like responses to the voltage steps

We next conducted a more detailed examination of the ion channel properties that allow channels to behave like inductors (Fig. 4). For this analysis, current responses to step voltage inputs were examined; these are often used to analyse the electrical properties of linear electrical circuits. We first theoretically verified the inductor currents in response to small square voltage steps (Fig. 4 *A*–*D*). In the parallel RLC circuit, the simulated inductor current exponentially increased and decreased at the onset and offset of a positive voltage step, and then reached individual steady‐state levels (Fig. 4 *C*).

We next examined the responses of ion channels in response to square voltage steps. Both 5 and 10 mV square voltage steps were applied from a holding potential of −30 mV, at which Kv11.3 was partially activated (Fig. 4 *E*–*H*). Kv11.3 currents were isolated by subtracting the trace recorded in the presence of E‐4031 from the trace recorded in the normal external solution. The application of E‐4031 completely inhibited both the current evoked by the voltage protocol for Kv11 and resonance in Kv11.3‐expressing cells (Fig. 4 *N*–*P*), thus confirming that the E‐4031‐sensitive Kv11 current is crucial for resonance. The Kv11.3 current exponentially increased and decreased from the baseline, then reached a steady state (Fig. 4 *E*–*H*). These properties were similar to those of the current flowing in the inductor in the theoretical analysis (Fig. 4 *C*).

We next examined whether the exponential increase or decrease of current in response to voltage steps reflected gradual activation or deactivation of Kv11.3 channels. If this were true, the Kv11.3 conductance at 5 or 10 mV depolarizing voltage steps would be larger than that at baseline (−30 mV). As expected, conductance at steady states (black lines in Fig. 4*I* and J) was increased by depolarization and decreased by hyperpolarization (Fig. 4 *I*–*K*), suggesting the participation of slow activation and deactivation of Kv11.3. In addition, we found another transient response. At the onset of depolarization, conductance transiently decreased relative to that at baseline (−30 mV) (arrowheads in Fig. 4 *I* and *J*). In contrast, conductance transiently increased at the offset of depolarization (Fig. 4 *I*, *J*, *L* and *M*). At the onset and offset of hyperpolarization, conductance increased and decreased (Fig. 4 *I*, *J*, *L* and *M*), respectively. Taken together, Kv11.3 conductance transiently decreased by depolarizing voltage changes and increased by hyperpolarizing voltage changes (Fig. 4 *L* and *M*). At around −30 mV, the K^+^ current flowing through Kv11.3 channels increased with depolarization. In this situation, such transient conductance changes oppose the current changes that are induced by voltage steps. This opposing conductance change probably has a similar influence on the flowing current as that of an inductor, and may potentially enable Kv11.3 to behave like the inductor.

### Heterologous expression of Kv11 channels generates membrane potential oscillation

Some recordings from Kv11‐expressing cells showed sustained membrane potential oscillation (Fig. 5), which was never observed in control HEK293 cells (Fig. 5 *A*). To determine the cells that had clear membrane potential oscillation, we classified all of the data regarding the peak power spectrum density (PSD) magnitude into two groups in a logarithmic scale using *k*‐means clustering (Fig. 5 *E*, right). We measured the frequency from the larger PSD group, which represented cells with clear membrane potential oscillation. The peak PSD of this group was larger than 10^−2.33^ V^2^ Hz^−1^. The peak PSD frequency of the Kv11.3‐expressing cells (2.7 ± 1.0 Hz, *n* = 8) was higher than that of Kv11.1(Merg1a) (0.8 ± 0.3 Hz, *n* = 16; one‐way ANOVA, *P* < 0.001; Holm–Šidák test, *P* < 0.001) or Kv11.1(Merg1b) (1.8 ± 0.3 Hz, *n* = 4; *P* = 0.017) (Fig. 5 *B*–*F*). The peak PSD frequency of Kv11.1(Merg1b) was higher than that of Kv11.1(Merg1a) (*P* = 0.009). We estimated the incidence of oscillation by calculating the percentage of these cells that had clear oscillation (Fig. 5 *G*). The estimated oscillation incidence tended to increase with depolarization, and the membrane potential dependencies of individual Kv11 channels were similar to those of Kv11 currents (Fig. 1 *I* and *J*).

Because a close relationship has been reported between resonant frequency and oscillation frequency (Hutcheon & Yarom, 2000; Erchova *et al*. 2004; Tohidi & Nadim, 2009), we examined their relationships in Kv11.1(Merg1b)‐ or Kv11.3‐expressing cells (Fig. 5 *H*–*K*). Because of technical limitations, resonant properties cannot be accurately assessed while oscillation is being generated (Hutcheon & Yarom, 2000), particularly at the same membrane potential. Therefore, frequencies at the largest PSD amplitude were compared with resonant frequencies at the largest resonant strength in individual cells, regardless of membrane potential. There was a significant correlation between peak PSD frequency and resonant frequency in both Kv11.3‐ (*r* = 0.75, *P* < 0.001, Pearson correlation coefficient) and Kv11.1(Merg1b)‐expressing cells (*r* = 0.87, *P* = 0.005) (Fig. 5 *H* and *I*). These results confirm a close relationship of the frequency properties between resonance and membrane potential oscillation. We also examined relationship between magnitudes of the resonant strength and the peak PSD. Resonant strength also correlated with peak PSD in Kv11.3‐expressing cells (*r* = 0.455, *P* = 0.019) (Fig. 5K), while the correlation coefficient is not large. The correlation in Kv11.1(Merg1b) was not statistically significant (*r* = 0.682, *P* = 0.063), but resonant strength tended to increase with the peak PSD in Kv11.1(Erg1b)‐expressing cells (Fig. 5 *J*).

Kv11.1(Merg1a)‐expressing cells did not show resonance when using the ZAP stimulus protocol (Fig. 2 *C*, *D* and *L*–*N*), although they exhibited clear membrane potential oscillation at the lower frequency range (Fig. 5 *B* and *E*–*G*). We assumed that such low‐frequency resonance was incorrectly assessed using the ZAP protocol because the frequency was close to the detection threshold (0.5 Hz). To examine resonance at a lower frequency range, sinusoidal currents with frequencies of 0.2, 0.4, 0.6, 0.8, 1.0, 1.2 and 2.0 Hz were individually applied to Kv11.1(Merg1a)‐expressing cells (Fig. 6 *A*), and magnitudes of output voltage were plotted against input current frequencies (Fig. 6 *B*–*D*). As a result, output voltage peaked around 0.8 Hz in a membrane potential‐dependent manner (Fig. 6 *D*), suggesting the occurrence of resonance at the low frequency range in Kv11.1(Merg1a)‐expressing cells. The resonant frequency was largely identical to the peak PSD frequency of Kv11.1(Merg1a)‐expressing cells, which confirms the relationship between resonance and peak PSD frequencies.

### Transfection of HCN1 induces resonance at hyperpolarized membrane potentials

It has been reported that resonance in many brain regions is dependent on HCN channels (Hutcheon & Yarom, 2000; Wang, 2010; Hashimoto, 2020). HCN1 is known to be a subtype that can induce resonance in neurons (Matsumoto‐Makidono *et al*. 2016; Hashimoto, 2020). However, it remains unclear whether transfection of HCN1 reproduces resonance. We therefore explored the influence of HCN1 transfection on resonance. The hyperpolarizing voltage protocol evoked HCN currents in HCN1‐transfected cells (Fig. 7 *A*, *B*, *D*), but not in control or Kv11‐expressing cells (Fig. 7 *A*, *E*–*G*). However, according to the aforementioned criteria for resonance generation, most HCN1‐expressing cells did not exhibit resonance at hyperpolarized potentials (Fig. 7 *H*, *I*, *M*–*O*). We assumed that the low incidence of resonance might have resulted from the low surface expression of HCN1, and thus tried to co‐transfect HCN1 with Rab8b‐interacting protein (TRIP8b, *Pex5l*) (Fig. 7 *J* and *K*). We used a splice variant of TRIP8b with an exon 1a followed by a subsequent variable exon 4 (1a−4), which has been reported to enhance the surface expression of HCN channels (Lewis *et al*. 2009; Santoro *et al*. 2009). Co‐expression with TRIP8b enhanced the HCN1 current (Fig. 7 *C* and *D*), and substantially increased the incidence of cells with resonance at hyperpolarized membrane potentials (8% to 31% at −60 mV and 15% to 20% at −75 mV, Fig. 7 *J*, *K*, *M*, *N*), although the incidence remained relatively low (Fig. 7 *O*). Similar to the Kv11.3 channel, the magnitude of the resonant strength did not show a significant correlation with HCN conductance (*r* = 0.11, *P* = 0.6, Pearson correlation coefficient) (Fig. 7 *L*). The *Z‐F* plot of HCN1‐ and TRIP8b‐expressing cells exhibiting resonance showed a hump (Fig. 7 *K*, orange) that peaked at 2.2 ± 0.6 Hz at −60 mV (*n* = 5) and 3.2 ± 0.9 Hz at −75 mV (*n* = 2). In all cells, the peak PSD was less than 10^−2.33^ V^2^ Hz^−1^, which suggests that membrane potential oscillation was not observed in HCN1‐expressing cells, probably because of the relatively weak resonant strength.

### Kv11.3 is crucial for resonance at depolarized membrane potentials in IO neurons

We finally examined the functional roles of Kv11 on resonance in neurons. The resonant frequency range of Kv11.1(Merg1a)‐expressing cells (0.6−0.8 Hz) (Fig. 6) was below the lower limit of resonant frequency in IO neurons (2−10 Hz) (Lampl & Yarom, 1997; Matsumoto‐Makidono *et al*. 2016), which suggests that Kv11.1(Merg1a) is not likely to be a candidate. In contrast, the resonant frequency range of Kv11.1(Merg1b)‐expressing cells (2−6 Hz) (Fig. 2 *J* and *M*) overlapped with those of IO neurons. However, Kv11.1(Merg1b)‐mediated resonance occurred from more depolarized membrane potentials (Fig. 2 *I*, *J*, *L*–*N*). Meanwhile, the frequency range of Kv11.3‐dependent resonance overlapped with that of IO neurons and began to be activated at more hyperpolarized potentials. These data suggest that Kv11.3 is likely to be the major subtype that generates resonance around the resting membrane potential in IO neurons. As already presented earlier in the results of the Kv11 current kinetics, the analysis of Kv11 currents also support this concept (Table 2).

**Figure 8:**
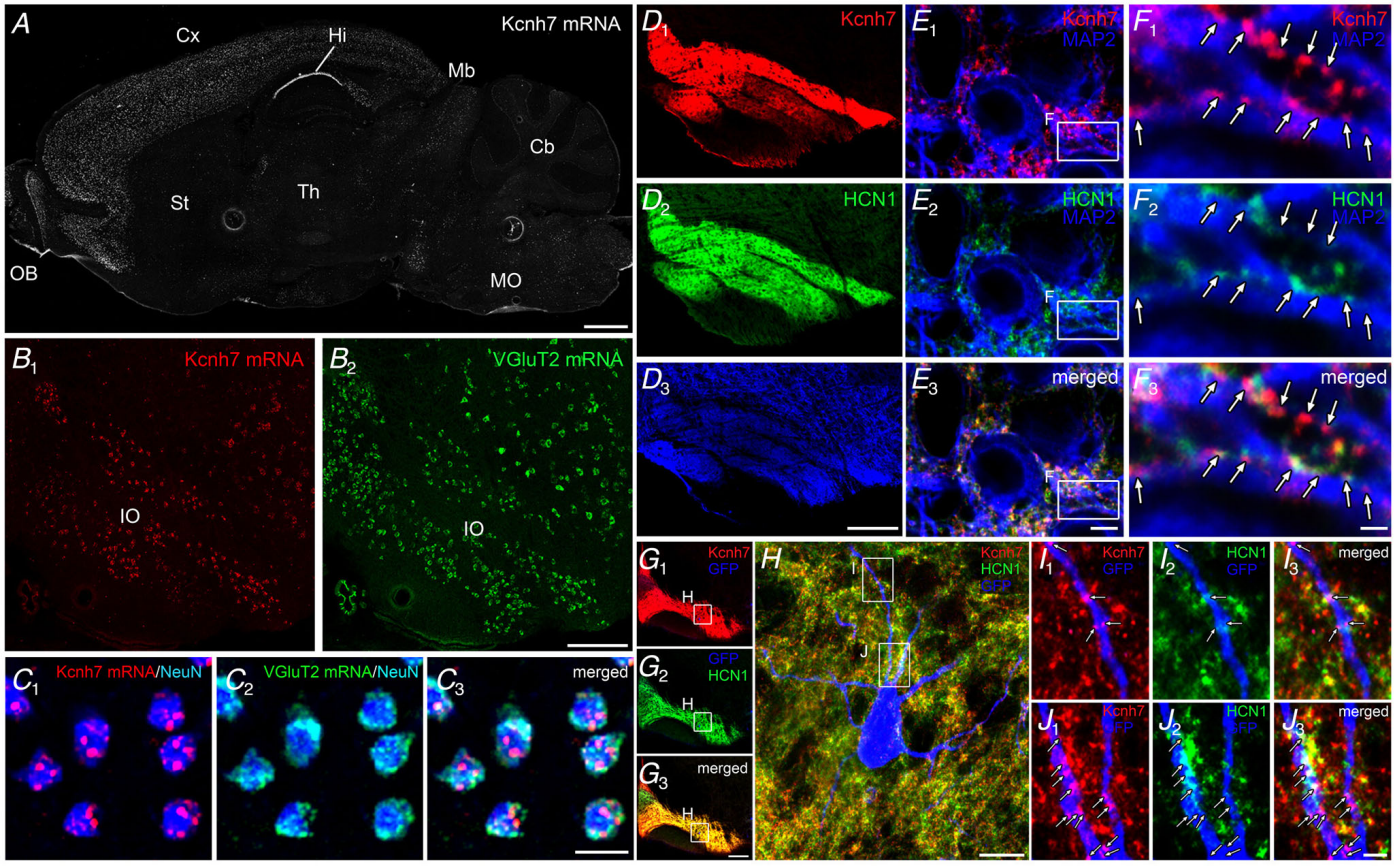
Punctate subcellular distribution of Kv11.3 on the dendrites of IO neurons *A*, fluorescent *in situ* hybridization of *Kcnh7* mRNA in the adult mouse brain. *B*, double‐labelling fluorescent *in situ* hybridization for *Kcnh7* (*B*_1_) and VGluT2 (*B*_2_) in the IO. *C*, higher magnification images of the IO. *Kcnh7* (red) and VGluT2 (green) mRNA was co‐expressed in IO neurons. *D*–*F*, triple immunofluorescence showing Kv11.3 (red) and HCN1 (green) localization on MAP2‐positive neurons (blue). Boxed areas in *E* are shown in *F*. Arrows indicate Kv11.3‐positive puncta. *G*–*J*, triple immunofluorescence showing Kv11.3 (red) and HCN1 (green) localization on a virally labelled GFP‐positive neuron (blue). The boxed area in *G* is shown in *H*. The boxed areas in *H* are enlarged in *I* and *J*. Arrows indicate Kv11.3‐positive puncta. Scale bars: *A*, 1 mm; *B*, *D*, *G*, 200 μm; *C*, 20 μm; *E*, 5 μm; *F*, *I*, *J*, 1 μm; *H*, 10 μm.

**Figure 9:**
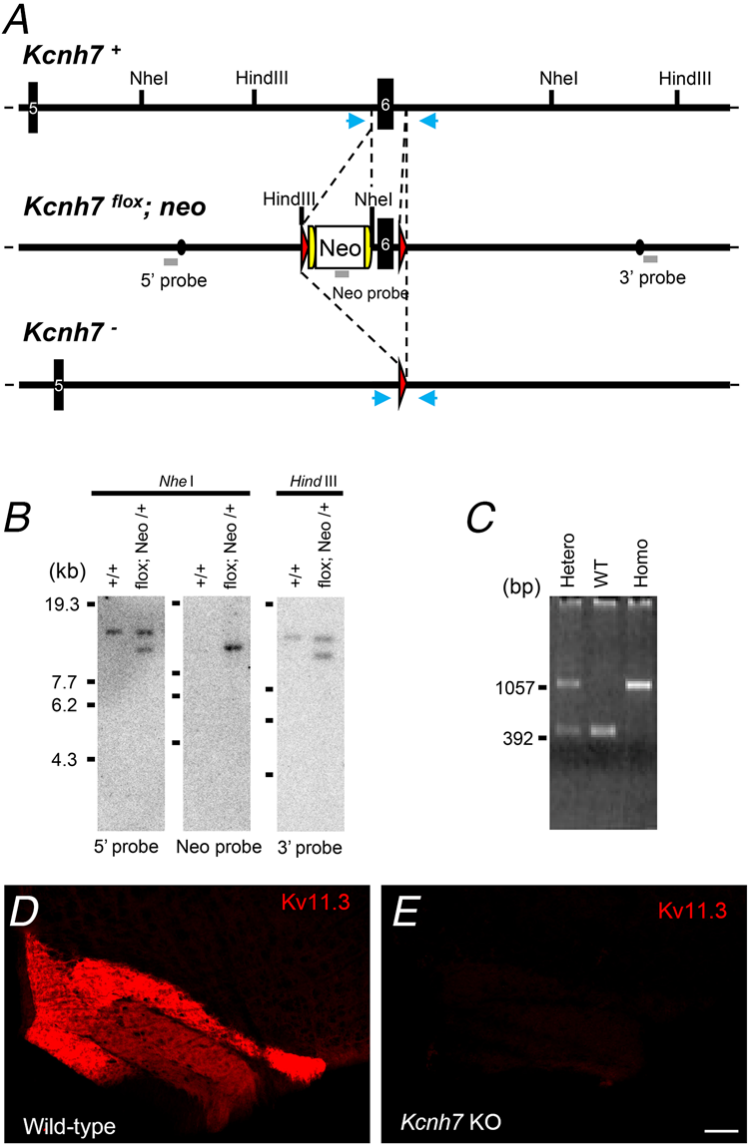
Generation of Kv11.3 KO mice *A*, schematic representation of the *Kcnh7* genomic DNA (*Kcnh7* +), targeted genome (*Kcnh7*‐flox; Neo) and targeted genome after Cre‐mediated recombination (*Kcnh7* –). Black numbered boxes indicate coding exons. Filled circles in the targeted genomes delineate the 5′ and 3′ termini of the targeting vectors. Grey bars indicate probe regions (5′, Neo and 3′) for Southern blot analysis. Two frt sequences (semicircles) were attached to remove the neomycin resistant gene (Neo). Triangles indicate loxP sequences. Arrows show primer positions for PCR genotyping. *B*, Southern blot analysis of wild‐type (*Kcnh7* +/+) and recombinant (*Kcnh7*‐flox; Neo/+) ES cell genomes. NheI‐digested DNA hybridized with the 5′ probe, 13.1 kb for the wild‐type and 10.3 kb for the targeted allele; NheI‐digested DNA hybridized with the Neo probe, 10.3 kb for the targeted allele; HindIII‐digested DNA hybridized with the 3′ probe, 11.8 kb for the wild‐type and 9.8 kb for the targeted allele. Positions of DNA size markers (kb) are indicated on the left. *C*, PCR genotyping of *Kcnh7* wild‐type (WT), KO heterozygote (Hetero), and homozygote (Homo) mice. A specific primer set produced a 1077 bp or 412 bp band in wild‐type or KO alleles, respectively. Positions of DNA size markers (bp) are indicated on the left. *D*, immunofluorescence showing Kv11.3 (red) expression in the IO. *E*, immunofluorescence showing almost no Kv11.3 expression in *Kcnh7* KO mice. Images were obtained at the same acquisition settings in *D*. Scale bar, 100 μm.

We confirmed Kv11.3 expression in IO neurons using histochemical methods (Fig. 8). *Kcnh7* transcripts were widely expressed in the adult mouse brain, including the IO (Fig. 8 *A* and *B*), as reported previously (Saganich *et al*. 2001; Perry & Sanguinetti, 2010). In the IO nucleus, *Kcnh7* mRNA was detected in VGluT2 mRNA‐expressing projection neurons (Fig. 8 *C*). The distribution of Kv11.3 protein in IO neurons was analysed using a specific antibody (Fig. 8 *D*–*J*). Kv11.3 immunoreactivity was distributed throughout the entire IO nucleus, but tended to be higher in the dorsal and medial accessory olive (Fig. 8 *D*_1_). The absence of immunolabelling in *Kcnh7* (Kv11.3) KO brains (Fig. 9 *D* and *E*) verified the specificity of Kv11.3 immunohistochemistry. Similar to HCN1 (Fig. 8*E* _2_ and *F*_2_) and Cav3.1 (Matsumoto‐Makidono *et al*. 2016), which are involved in resonance in IO neurons, Kv11.3 immunoreactivity appeared as punctate labelling on MAP2‐positive dendrites of IO neurons (Fig. 8*E* _1_ and *F*_1_). Clusters of Kv11.3 and HCN1 were closely apposed and partially overlapped (Fig. *8E* _3_ and *F*_3_). This spatial relationship between Kv11.3‐ and HCN1‐positive puncta was also confirmed in a GFP‐labelled single neuron (Fig. 8*G*–*J*).

**Figure 10:**
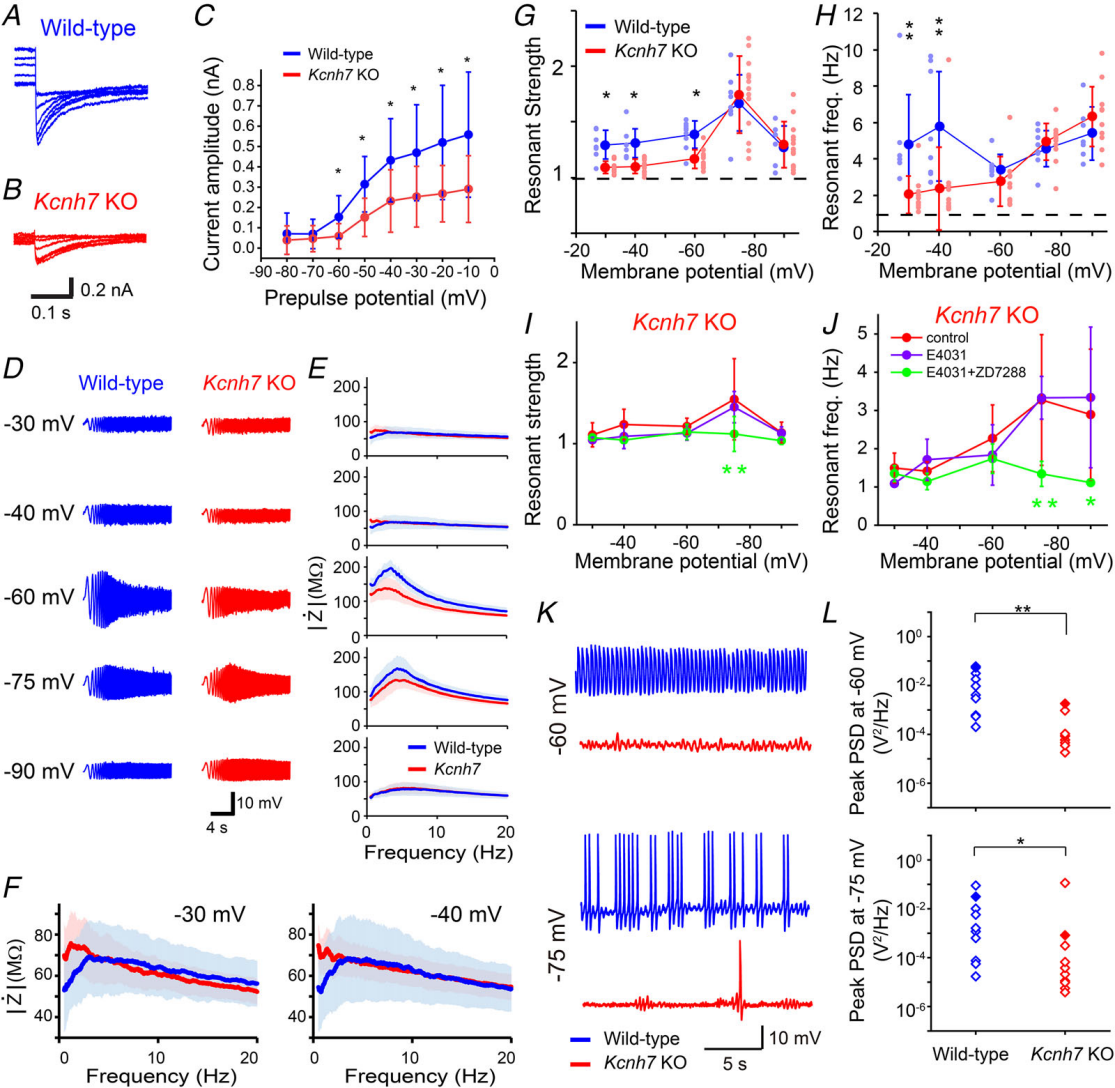
Kv11 current and resonance in IO neurons are impaired in Kv11.3 KO mice *A*, representative current traces of Kv11 tail currents recorded from a wild‐type IO neuron in high K^+^ external solution. Currents were evoked by the same protocol shown in Fig. 1 *A*. Current traces were calculated by subtracting traces in the presence of E‐4031 from those recorded in control external solution. *B*, representative current traces of a *Kcnh7* KO IO neuron. *C*, the plots of tail current amplitudes from wild‐type (blue, 9 cells, six animals) and *Kcnh7* KO (red, 8 cells, four animals) neurons. Data are presented as the means ± SD. *D*, representative voltage waves of a wild‐type (left) or a *Kcnh7* KO (right) IO neuron in response to chirp currents (±50 pA) at membrane potentials of −30, −40, −60, −75 and −90 mV in the Ca^2+^‐free external solution. *E*, average *Z‐F* profiles of IO neurons in wild‐type (blue, 7−9 cells, three animals) and *Kcnh7* KO (red, 10−13 cells, three animals) mice. Shaded area indicates the mean ± 95% confidence interval. *F*, magnified graphs of −30 mV (upper) and −40 mV (lower) from *E*. *G* and *H*, membrane potential dependency of resonant strength (*G*) and resonant frequency (*H*) of IO neurons in wild‐type (7−9 cells, three animals) and Kv11.3 KO (10−13 cells, three animals) mice. Data are presented as the means ± SD. ^*^ *P* < 0.05; ^**^ *P* < 0.01 (two‐way ANOVA, *post hoc*, Holm–Šidák test). *I* and *J*, pharmacological effects of E‐4031 (10 μm) and E‐4031 + ZD7288 (20 μm) on the membrane potential dependency of resonant strength (*I*) and resonant frequency (*J*) in *Kcnh7* KO mice (control, *n* = 5−7, 3 animals; E‐4031, *n* = 6, 2 animals; E‐4031 + ZD7288, *n* = 4, 3 animals). Data are presented as the means ± SD. Statistical differences of E‐4031 and E‐4031 + ZD7288 from the control are presented. ^*^ *P* < 0.05; ^**^ *P* < 0.01, two‐way ANOVA, *post hoc*, Holm–Šidák test. *K*, representative traces of STO in wild‐type (blue) and *Kcnh7* (red) KO IO neurons at −60 mV (upper) and −75 mV (lower). *L*, peak PSD magnitudes of STO at −60 mV (upper) and −75 mV (lower) in wild‐type (*n* = 10, 1 animals) and *Kcnh7* KO (*n* = 10, 3 animals) IO neurons. Data are presented as the means ± SD. ^*^ *P* < 0.05; ^**^ *P* < 0.01, Mann–Whitney *U* test.

We also examined the functional roles of Kv11.3 in IO neurons using *Kcnh7* KO mice (Fig. 9). The voltage‐dependent, E‐4031‐sensitive current was significantly decreased in *Kcnh7* KO mice (Fig. 10 *A*–*C*). To record resonance, Ca^2+^‐free external solution was used to suppress Cav3.1‐dependent amplifying conductance (Matsumoto‐Makidono *et al*. 2016). In wild‐type mice, the resonant strength increased with hyperpolarization of the membrane potential, peaked at −75 mV, and then declined with further hyperpolarization (Fig. 10 *D*–*H*) (Matsumoto‐Makidono *et al*. 2016). Notably, the *Z‐F* profile at −30 and −40 mV showed a clear hump (Fig. 10 *E* and *F*) and the resonant frequency was around 5 Hz (Fig. 10 *H*), suggesting the generation of resonance. In *Kcnh7* KO mice, the resonant strength approached 1 and the resonant frequency shifted towards lower frequencies at −30, −40, and −60 mV, but not at −75 or −90 mV (Fig. 10 *G* and *H*). Importantly, the *Z‐F* profiles at −30 and −40 mV exhibited a monotonic decline with frequency (Fig. 10 *F*, red line). These data suggest the loss of an inductor‐like factor at depolarized potentials in IO neurons in *Kcnh7* KO mice. The *Z‐F* plot of *Kcnh7* KO mice was not affected by E‐4031, suggesting that the contributions of other Kv11 subtypes were negligible (Fig. 10 *I* and *J*). The subsequent application of ZD7288 (20 μm), a blocker for HCN channels, completely blocked resonance at hyperpolarized potentials in *Kcnh7* KO mice (Fig. 10 *I* and *J*). Kv11.3 appears to be a crucial subtype for the generation of resonance around the resting membrane potential (Khosrovani *et al*. 2007) in IO neurons.

Finally, we examined the influence of *Kcnh7* KO on STO. STO was recorded in normal external solution by 10 min after whole‐cell recording began because STO tended to disappear. All peak PSDs were clustered into two groups using *k*‐means clustering, with a threshold of 10^−3.27^ V^2^ Hz^−1^. Neurons in the larger peak PSD group were taken as neurons with clear oscillation. STO was observed in 70%−90% of wild‐type IO neurons (9/10 at −60 mV and 7/10 at −75 mV) at membrane potentials of −75 and −60 mV (Fig. 10 *K* and *L*). However, compared with the wild‐type results, STO incidence was lower in the *Kcnh7* KO mice (2/10 at −60 mV and 2/10 at −75 mV) (Fig. 10 *K* and *L*). Furthermore, the peak PSD was significantly lower in *Kcnh7* KO mice (Fig. 10 *L*). The peak PSD frequencies of the larger peak PSD group were from 1.1 to 5.0 Hz (2.5 ± 0.7 Hz at −60 mV (*n *= 9, 1 animal); 2.9 ± 0.9 Hz at −75 mV (*n* = 7, 1 animal)) in wild‐type and from 2.1 to 4.2 Hz (3.1 ± 1.4 Hz at −60 mV (*n* = 2, 1 animal); 3.5 ± 0.9 Hz at −75 mV (*n = *2, 2 animals)) in *Kcnh7* KO mice. These results suggest that STO around the resting membrane potential is also critically dependent on Kv11.3 in IO neurons.

## Discussion

### Kv11.1(Merg1a), Kv11.1(Merg1b), and Kv11.3 channels induce resonance

In the present study, heterologous expression of Kv11 channels reproduced resonance in HEK293 cells (Figs 2 and 6). Kv11.3 has properties that are crucial for inductor‐like response (Figs 3 and 4). The inductor in an electrical circuit generates voltage with a polarity that opposes the current change. Furthermore, the magnitude of the opposing voltage linearly increases with the rate of current change (V(t)=L(dI(t)/dt), *L*: inductance). Therefore, rapid current changes caused by the onset and offset of the voltage step are preferentially suppressed. Consequently, currents with lower frequencies pass through the inductor (low‐pass filter). Because ion channels do not generate such opposing voltages, their mechanisms for generating inductor‐like responses should be different. The analysis of current responses to voltage steps suggests that the inductor‐like responses in the present study were mediated by the following two processes: the fast opposing conductance change just after the voltage onset, and the subsequent slow activation or deactivation of ion channels (Fig. 4). At −30 mV, a part of Kv11.3 is persistently activated. If open Kv11.3 channels acted as an ideal conductance, the current would show a stepwise increase from the baseline current at the onset of the 5 or 10 mV voltage step, according to Ohm's law. However, the step current increase is likely to be cancelled by the fast opposing conductance decrease, and the subsequent transient response starts from around the baseline. The fast opposing conductance decrease or increase may have similar influences, suppressing transient current via the opposing potential generation by the inductor. The opposing conductance change may be caused by inactivation of Kv11.3. It has been reported that inactivation and recovery from inactivation of Kv11 channels occurs very quickly (Perry & Sanguinetti, 2010; Gustina & Trudeau, 2012; Bauer & Schwarz, 2018). Continuously activating Kv11.3 around −30 mV might cause weak inactivation, and a small amount of depolarization may quickly cause further Kv11.3 inactivation. At hyperpolarization, the opposite changes would be expected to occur. Currently, we cannot completely rule out the possibility that unknown auxiliary factors necessary for resonance are intrinsically expressed in HEK293 cells. However, the generation of resonance is unlikely to be affected by the unexpected overexpression of other voltage‐dependent ion channels, because the voltage protocol for the HCN current did not evoke detectable currents (Fig. 7 *A*, *E*–*G*), and E‐4031 largely blocked the current evoked by membrane potential depolarizations (Fig. 4 *N*).

It has been reported that resonance in neurons is mediated by several types of K^+^ channels, such as the M‐type K^+^ channel (Hu *et al*. 2002; Peters *et al*. 2005; Heys *et al*. 2010; Boehlen *et al*. 2013; Vera *et al*. 2014; Honigsperger *et al*. 2015) and SK channels (Xue *et al*. 2012). However, it is unclear whether or not these candidate channels share common properties to behave like inductors. Analysis of these candidate K^+^ channels will provide more information about the crucial gating properties for responding like inductor.

### Relationships between resonance and oscillation

In the present study, resonant frequency strongly correlated with peak PSD frequency in Kv11‐expressing cells (Fig. 5 *H* and *I*). These data confirm the close relationship of frequency properties between resonance and membrane potential oscillation (Hutcheon & Yarom, 2000; Erchova *et al*. 2004; Tohidi & Nadim, 2009). According to previous theoretical and experimental analyses, membrane potential oscillation requires interactions between two or more ion channels with distinct gating properties and ion permeabilities (Alonso & Llinas, 1989; McCormick & Pape, 1990; Fransen *et al*. 2004; Wang, 2010; Chen *et al*. 2018). Therefore, from the present analysis alone, we are unable to conclude that stable membrane potential oscillation can be induced solely by Kv11 expression. However, the kinetics of Kv11 channels may affect the frequency of oscillation. The peak PSD frequency in Kv11.1(Merg1a) was substantially slower than that of Kv11.1(Merg1b) and Kv11.3 (Fig. 5). Because the activation and deactivation kinetics of Kv11.1(Merg1a) are also slower than those of other subtypes (Fig. 1 *F*–*H*) (London *et al*. 1997; Shi *et al*. 1997; Schledermann *et al*. 2001; Wimmers *et al*. 2002; Hirdes *et al*. 2005; Sturm *et al*. 2005; Niculescu *et al*. 2013), there may be a relationship between the kinetics of Kv11 and the frequency of oscillation.

### HCN1 generates resonance at hyperpolarized potentials

HCN channels reportedly have sufficient properties to act as resonating conductance (Narayanan & Johnston, 2008; Matsumoto‐Makidono *et al*. 2016; Hashimoto, 2020) and play a pivotal role in generating resonance at hyperpolarized potentials in many neuronal systems (Hutcheon & Yarom, 2000; Wang, 2010; Hashimoto, 2020). Both Kv11 and HCN channels are known to be involved in the CNBD cation channel family, which is part of the voltage‐dependent K^+^ channel superfamily (James & Zagotta, 2018). These channels are characterized by a cyclic nucleotide‐binding homology domain (CNBHD) in the C‐terminal region (Perry & Sanguinetti, 2010; Gustina & Trudeau, 2012; Morais‐Cabral & Robertson, 2015; Bauer & Schwarz, 2018). The present study raises the possibility that these channels share not only structural similarities, but also functional similarities for membrane potential rhythmogenesis.

We have previously reported that HCN1 plays a role as the inductor in resonance in IO neurons (Matsumoto‐Makidono *et al*. 2016). In the present study, some cells transfected with HCN1 induced resonance at hyperpolarized potentials (Fig. 7 *M*–*O*). Unexpectedly, the incidence of HCN1‐expressing cells with resonance was relatively low and the resonant strength was weak in the present study (Fig. 7 *H*–*K*, *M*–*O*). Although we cannot rule out the possibility that the membrane expression of HCN1 was too low to induce resonance, HCN1 might require additional factors to induce clear resonance. The overall *Z‐F* profile of cells without resonance in HCN1 & TRIP8b expressing cells (the grey line in Fig. 7 *K*) was comparable to those of control cells at −30 mV (Fig. 2 *B*), but was significantly lower at −75 mV. This suggests that HCN1 conductance is persistently activated by hyperpolarization without exhibiting frequency‐dependent impedance changes. The expression of HCN1 alone may be insufficient to induce clear resonance. This concept is supported by previous reports demonstrating that co‐expression of HCN channels and Kir2.1 is necessary to reproduce spontaneous slow oscillation (Sun *et al*. 2017; Chen *et al*. 2018).

### Roles of Kv11 channels in IO neurons

Both Kv11.3 and Kv11.1 mRNA are expressed in the IO nucleus (Shi *et al*. 1997; Saganich *et al*. 2001; Perry & Sanguinetti, 2010), and the E‐4031‐sensitive Kv11 current remained in *Kcnh7* KO mice (Fig. 10 *A*–*C*), which suggests the functional expression of Kv11.1 in IO neurons. However, resonance at membrane potentials ranging from −60 to −30 mV was largely mediated by Kv11.3 in IO neurons (Fig. 10 *D*–*J*). Analysis of Kv11.1 kinetics suggested that the major splice variant was likely to be Merg1b, which was activated by strong membrane potential depolarization (Fig. 1 *I* and *J* and Table 1) (Hirdes *et al*. 2005; Niculescu *et al*. 2013). Kv11.1 may be activated by stronger depolarizations in IO neurons, and may play a role in accommodating action potential firing rather than in generating resonance around the resting membrane potential (Bauer & Schwarz, 2018).

The voltage range of Kv11.3‐dependent resonance largely overlaps with the resting membrane potential in IO neurons *in vivo* (−45 to −65 mV) (Khosrovani *et al*. 2007), which suggests that Kv11.3 is a crucial subtype for generating resonance at the resting membrane potential. The resonant strength at −30 to −60 mV was weaker than that at more hyperpolarized potentials (Fig. 10 *G*). However, such weak resonance may also promote the frequency response, because the activity of resonating conductance is normally enhanced by amplifying conductance (Hutcheon & Yarom, 2000). Because IO neurons are also known to be connected by gap junctions (Devor & Yarom, 2002), Kv11.3‐dependent resonance may contribute to facilitating synchronized activation at the resting membrane potential among interconnected IO neurons. Furthermore, the present study suggests that Kv11.3‐dependent resonance contributes to the generation of STO in IO neurons (Fig. 10 *K* and *L*). Previous analyses have suggested that STO in IO neurons shows the largest amplitude at around −60 mV, and it is also observed at more depolarized membrane potentials (Benardo & Foster, 1986; Bleasel & Pettigrew, 1994; Lampl & Yarom, 1997; Leznik & Llinas, 2005; Choi *et al*. 2010). As mentioned earlier in the discussion of the relationship between resonance and oscillation, we cannot currently conclude that Kv11.3 solely induces oscillation. However, Kv11.3 may be one of crucial ion channels to induce STO at the resting membrane potential in IO neurons.

Many previous studies have reported that resonance at depolarized and hyperpolarized potentials is mediated by different ion channels (Gutfreund *et al*. 1995; Hu *et al*. 2002; Heys *et al*. 2010; Xue *et al*. 2012; Boehlen *et al*. 2013; Vera *et al*. 2014). We previously reported that resonance from −60 to −90 mV in the hyperpolarized potential range was mediated by the HCN1 channel in IO neurons (Matsumoto‐Makidono *et al*. 2016). The present analysis suggests that Kv11.3 and HCN1 function as resonating conductances at depolarized and hyperpolarized membrane potentials, respectively, in IO neurons (Fig. 10 *I* and *J*). The voltage ranges for HCN1 and Kv11.3 partially overlap, and resonance at −60 mV is suppressed by both E‐4031 and ZD7288, a blocker for HCN channels (Matsumoto‐Makidono *et al*. 2016). The resonance at −60 mV is also suppressed both in *Kcnh7* and HCN1 KO mice (Fig. 10 *D*–*H*) (Matsumoto‐Makidono *et al*. 2016). Because the membrane potential of −60 mV is near the border of the activation thresholds for Kv11.3 and HCN1, activation of both channels might be required to ensure the generation of sufficient resonance.

## Additional information

### Competing interests

The authors declare no competing financial interests.

### Author contributions

T.M. and K.H. designed the study. T.M., M.Y., M.A. and Y.M. performed experiments. T.M., M.Y., M.A., Y.M., H.M., H.K., K.S., M.W. and K.H. analysed the data. M.Y., M.A., Y.M., H.M., H.K., K.S. and M.W. contributed reagents/analysis tools. T.M., M.Y., M.A., Y.M., H.M., H.K., K.S., M.W. and K.H. wrote the paper. All authors approved the final version of the article and agree to be accountable for all aspects of the work in ensuring that questions related to the accuracy or integrity of any part of the work are appropriately investigated and resolved. All persons designated as authors qualify for authorship, and all those who qualify for authorship are listed.

### Funding

This work was supported by Grants‐in Aid for Scientific Research (17H06313 and 17K08503 to M.Y., 16J40095 and 19K07994 to Y.M., 17H03551 and 18H04947 to K.H.) from the Ministry of Education, Culture, Sports, Science and Technology of Japan. This work was also supported by the Strategic Research Program for Brain Sciences (17dm0107093h0002) from AMED to K.H., and The Takeda Science Foundation to H.K. Generation of the anti‐Kv11.3 antibody was supported by JSPS KAKENHI ‘Advanced Bioimaging Support’ (JP16H06280). Generation of the *Kcnh7* KO mice was supported by JSPS KAKENHI ‘Advanced Animal Model Support’ (JP16H06276).

## Supporting information


**Statistical Summary Document**

## Data Availability

The data that support the findings of this study are available from the corresponding author upon reasonable request.
