# Supplementary material for: Kv11 (ether‐à‐go‐go‐related gene) voltage‐dependent K+ channels promote resonance and oscillation of subthreshold membrane potentials
Source: J Physiol. 2020 Nov 18;599(2):547–69. doi: 10.1113/JP280342 (PMC7839749; doi:10.1113/JP280342)
Supplement: Supplementary file 1 — Statistical Summary Document [file TJP-599-547-s001.docx]

**Manuscript Title:** Kv11 (ether-à-go-go-related gene) voltage-dependent K^+^ channels promote resonance and oscillation of subthreshold membrane potential

**Authors:** Toshinori Matsuoka, Miwako Yamasaki, Manabu Abe, Yukiko Matsuda, Hiroyuki Morino, Hideshi Kawakami, Kenji Sakimura, Masahiko Watanabe and Kouichi Hashimoto

**Animal model used:** Mouse – C57BL/6J and *Kcnh7* KO on C57BL/6N background.

**Underlying hypothesis:** This investigation tests the hypothesis that Kv11 channels mediate resonance and oscillation of the membrane potential in inferior olivary (IO) neurons.

**Definitions of ‘n’:** n = individual HEK293 cells or IO neurons. IO neurons were recorded from brain slices prepared from the mouse medulla oblongata. Number of mice are stated under the number of neurons.

**Statistical summary table:**

| Experimental question number* | Finding/ conclusion | Experimental location/ variable  e.g. muscle, neocortex or genotype | Mean value  (or other summary statistic) | SD | n val. | P** | Units | Data comparisons  e.g. WT vs KO | Statistical test | Any other variable  e.g. subjects’ age or sex | Figure/ table in which data are presented | Comments  e.g. observation |
| --- | --- | --- | --- | --- | --- | --- | --- | --- | --- | --- | --- | --- |
| 1. Resonant strength in human embryonic kidney 293 (HEK293) cells transfected with Kv11 channels | Resonant strength of Kv11.1(murine ether-à-go-go-related gene (Merg)1b)-expressing HEK293 cells is higher than that of non-transfected HEK293 cells at -10mV; Resonant strength of Kv11.3-expressing HEK293 cells is higher than that of non-transfected HEK293 cells at-30mV and -40mV; Resonant strength of Kv11.1 (Merg1a)-expressing HEK293 cells is identical to that of non-transfected HEK293 cells. | Control HEK293 cells at -10mV | 1.041 | 0.062 | 6 | – | fold (ratio of the peak impedance relative to impedance at 0.5Hz) | Control vs x | One way ANOVA with  Holm–Šidák test |  | 2I | -10 mV |
|  |  | Merg1b at -10mV | 1.466 | 0.398 | 7 | **0.026** | fold | x |  |  |  |  |
|  |  | Control at -20mV | 1.049 | 0.063 | 10 | 0.187 | fold |  | One way ANOVA |  |  | -20mV |
|  |  | Merg1a at -20mV | 1.010 | 0.008 | 3 |  | fold |  |  |  |  |  |
|  |  | Merg1b at -20mV | 1.372 | 0.614 | 8 |  | fold |  |  |  |  |  |
|  |  | Control at -30mV | 1.077 | 0.090 | 11 | – | fold | Control vs y | One way ANOVA with  Holm–Šidák test |  |  | -30mV |
|  |  | Merg1a at -30mV | 1.012 | 0.028 | 8 | 0.908 | fold | y |  |  |  |  |
|  |  | Merg1b at -30mV | 1.181 | 0.367 | 8 | 0.978 | fold | y |  |  |  |  |
|  |  | Kv11.3 at -30mV | 2.303 | 1.607 | 30 | **0.016** | fold | y |  |  |  |  |
|  |  | Control at -40mV | 1.052 | 0.030 | 11 | – | fold | Control vs z | One way ANOVA with  Holm–Šidák test |  |  | -40mV |
|  |  | Merg1a at -40mV | 1.003 | 0.006 | 8 | 0.984 | fold | z |  |  |  |  |
|  |  | Merg1b at -40mV | 1.042 | 0.055 | 8 | 0.975 | fold | z |  |  |  |  |
|  |  | Kv11.3 at -40mV | 1.812 | 0.880 | 35 | **0.006** | fold | z |  |  |  |  |
|  |  | Control at -60mV | 1.039 | 0.065 | 11 | 0.611 | fold |  | One way ANOVA |  |  | -60mV |
|  |  | Merg1a at -60mV | 1.174 | 0.112 | 8 |  | fold |  |  |  |  |  |
|  |  | Merg1b at -60mV | 1.062 | 0.061 | 8 |  | fold |  |  |  |  |  |
|  |  | Kv11.3 at -60mV | 1.099 | 0.289 | 35 |  | fold |  |  |  |  |  |
|  |  | Control at -75mV | 1.041 | 0.068 | 10 | 0.724 | fold |  | One way ANOVA |  |  | -75mV |
|  |  | Merg1a at -75mV | 1.030 | 0.042 | 2 |  | fold |  |  |  |  |  |
|  |  | Kv11.3 at -75mV | 1.059 | 0.068 | 16 |  | fold |  |  |  |  |  |
| 2. Resonant frequency in HEK293 cells transfected with Kv11 | Resonant frequency of Kv11.1(Merg1b)-expressing HEK293 cells is higher than that of non-transfected HEK293 cells at -10mV and -20mV; Resonant frequency of Kv11.3-expressing HEK293 cells is higher than that of non-transfected HEK293 cells at -30mV and -40mV;  Resonant frequency of Merg1a-expressing HEK293 cells is identical to that of non-transfected HEK293 cells. | Control HEK293 cells at -10mV | 0.905 | 0.527 | 6 | – | Hz | Control vs x | One way ANOVA with  Holm–Šidák test |  | 2J | -10mV |
|  |  | Merg1b at -10mV | 2.123 | 1.221 | 7 | **0.045** | Hz | x |  |  |  |  |
|  |  | Control at -20mV | 0.836 | 0.253 | 10 | – | Hz | Control vs y | One way ANOVA with  Holm–Šidák test |  |  | -20mV |
|  |  | Merg1a at -20mV | 0.600 | 0.088 | 3 | 0.639 | Hz | y |  |  |  |  |
|  |  | Merg1b at -20mV | 2.502 | 1.168 | 8 | **<0.001** | Hz | y |  |  |  |  |
|  |  | Control at -30mV | 0.871 | 0.259 | 11 | – | Hz | Control vs z | One way ANOVA with  Holm–Šidák test |  |  | -30mV |
|  |  | Merg1a at -30mV | 0.561 | 0.095 | 8 | 0.508 | Hz | z |  |  |  |  |
|  |  | Merg1b at -30mV | 1.369 | 1.234 | 8 | 0.495 | Hz | z |  |  |  |  |
|  |  | Kv11.3 at -30mV | 2.293 | 1.201 | 30 | **<0.001** | Hz | z |  |  |  |  |
|  |  | Control at -40mV | 0.913 | 0.271 | 11 | – | Hz | Control vs xx | One way ANOVA with  Holm–Šidák test |  |  | -40mV |
|  |  | Merg1a at -40mV | 0.530 | 0.016 | 8 | 0.76 | Hz | xx |  |  |  |  |
|  |  | Merg1b at -40mV | 1.072 | 1.054 | 8 | 0.784 | Hz | xx |  |  |  |  |
|  |  | Kv11.3 at -40mV | 2.386 | 1.543 | 35 | **0.003** | Hz | xx |  |  |  |  |
|  |  | Control at -60mV | 0.716 | 0.229 | 11 | 0.359 | Hz |  | One way ANOVA |  |  | -60mV |
|  |  | Merg1a at -60mV | 1.049 | 0.065 | 8 |  | Hz |  |  |  |  |  |
|  |  | Merg1b at -60mV | 0.721 | 0.229 | 8 |  | Hz |  |  |  |  |  |
|  |  | Kv11.3 at -60mV | 1.281 | 1.416 | 35 |  | Hz |  |  |  |  |  |
|  |  | Control at -75mV | 0.760 | 0.328 | 10 | 0.543 | Hz |  | One way ANOVA |  |  | -75mV |
|  |  | Merg1a at -75mV | 0.687 | 0.237 | 2 |  | Hz |  |  |  |  |  |
|  |  | Kv11.3 at -75mV | 1.202 | 1.350 | 16 |  | Hz |  |  |  |  |  |
| 3. Relationship between conductance and the Maximum (max.) resonant strength in Merg1b-expressing cells | There is no significant correlation. | Merg1b-expressing HEK293 cells | r = 0.295 |  | 8 | 0.478 | nS (conductance) ; fold (resonant strength) | Merg1b Conductance vs Max. Resonant strength | Pearson’s product moment correlation |  | 2K |  |
| 4. Relationship between conductance and the max. resonant strength in Kv11.3-expressing cells | There is no significant correlation. | Kv11.3-expressing HEK293 cells | r = 0.144 |  | 18 | 0.567 | nS (conductance); fold (resonant strength) | Kv11.3 Conductance vs Max. Resonant strength | Pearson’s product moment correlation |  | 2K |  |
| 5. Frequency-dependence of sinusoidal current amplitudes in control and E-4031 solutions in Kv11.3-expressing cells | Amplitude of currents in the control solution is larger than that in the E-4031 solution at 0.5 Hz. | Control at 0.5 Hz | 338.3 | 191.9 | 5 | – | pA | Control vs E-4031 at 0.5 Hz | Two-way ANOVA  Holm–Šidák test |  | 3F | 0.5Hz |
|  |  | E-4031 at 0.5 Hz | 135.7 | 55.1 | 5 | **0.001** | pA |  |  |  |  |  |
|  |  | Control at 1.0 Hz | 227.2 | 122.1 | 5 | – | pA | Control vs E-4031 at 1.0 Hz |  |  |  | 1.0Hz |
|  |  | E-4031 at 1.0 Hz | 136.5 | 57.7 | 5 | 0.132 | pA |  |  |  |  |  |
|  |  | Control at 3.0 Hz | 128.0 | 66.3 | 5 | – | pA | Control vs E-4031 at 3.0 Hz |  |  |  | 3.0Hz |
|  |  | E-4031 at 3.0 Hz | 146.2 | 63.8 | 5 | 0.759 | pA |  |  |  |  |  |
|  |  | Control at 5.0 Hz | 146.9 | 66.4 | 5 | – | pA | Control vs E-4031 at 5.0 Hz |  |  |  | 5.0Hz |
|  |  | E-4031 at 5.0 Hz | 152.9 | 67.0 | 5 | 0.920 | pA |  |  |  |  |  |
|  |  | Control at 10 Hz | 207.1 | 81.1 | 5 | – | pA | Control vs E-4031 at 10 Hz |  |  |  | 10Hz |
|  |  | E-4031 at 10 Hz | 164.3 | 71.8 | 5 | 0.473 | pA |  |  |  |  |  |
| 6. Frequency-dependence of E-4031-sensitive current in Kv11.3-expressing cells | E-4031-sensitive current decreases with increase in input voltage frequency. | 0.5Hz | 369.1 | 250.3 | 5 | 0.158 | pA | 0.5Hz vs 1.0Hz | One way ANOVA with  Holm–Šidák test |  | 3G |  |
|  |  | 1.0Hz | 176.4 | 116.0 | 5 | 0.643 | pA | 1.0Hz vs 3.0Hz |  |  |  |  |
|  |  | 3.0Hz | 68.1 | 33.6 | 5 | 0.977 | pA | 3.0Hz vs 5.0Hz |  |  |  |  |
|  |  | 5.0Hz | 52.9 | 16.5 | 5 | 0.986 | pA | 5.0Hz vs 10Hz |  |  |  |  |
|  |  | 10Hz | 77.4 | 26.4 | 5 | **0.012** | pA | 10Hz vs 0.5Hz |  |  |  |  |
|  |  |  |  |  |  | **0.010** |  | 0.5Hz vs 3.0Hz |  |  |  |  |
|  |  |  |  |  |  | **0.007** |  | 0.5Hz vs 5.0Hz |  |  |  |  |
|  |  |  |  |  |  | 0.640 |  | 10Hz vs 1.0Hz |  |  |  |  |
|  |  |  |  |  |  | 0.578 |  | 1.0Hz vs 5.0Hz |  |  |  |  |
|  |  |  |  |  |  | 0.907 |  | 3.0Hz vs 10Hz |  |  |  |  |
| 7. Frequency-dependent phase shifts in control and E-4031 solutions in Kv11.3-expressing cells | E-4031-sensitive conductance causes a large phase lag at 0.5 and 1.0 Hz. | Control at 0.5 Hz | -0.758 | 0.235 | 5 | – | radian | Control vs E-4031 at 0.5 Hz | Two-way ANOVA  Holm–Šidák test |  | 3J | 0.5Hz |
|  |  | E-4031 at 0.5 Hz | 0.041 | 0.006 | 5 | **<0.001** | radian |  |  |  |  |  |
|  |  | Control at 1.0 Hz | -0.789 | 0.304 | 5 | – | radian | Control vs E-4031 at 1.0 Hz |  |  |  | 1.0Hz |
|  |  | E-4031 at 1.0 Hz | 0.077 | 0.016 | 5 | **<0.001** | radian |  |  |  |  |  |
|  |  | Control at 3.0 Hz | -0.121 | 0.168 | 5 | – | radian | Control vs E-4031 at 3.0 Hz |  |  |  | 3.0Hz |
|  |  | E-4031 at 3.0 Hz | 0.133 | 0.047 | 5 | **0.027** | radian |  |  |  |  |  |
|  |  | Control at 5.0 Hz | 0.403 | 0.291 | 5 | – | radian | Control vs E-4031 at 5.0 Hz |  |  |  | 5.0Hz |
|  |  | E-4031 at 5.0 Hz | 0.150 | 0.057 | 5 | **0.028** | radian |  |  |  |  |  |
|  |  | Control at 10 Hz | 0.518 | 0.194 | 5 | – | radian | Control vs E-4031 at 10 Hz |  |  |  | 10Hz |
|  |  | E-4031 at 10 Hz | 0.165 | 0.057 | 5 | **0.003** | radian |  |  |  |  |  |
| 8. Steady-state Kv11.3 conductance at different membrane potentials | Steady-state conductance increases with depolarizations | Conductance at -40 mV relative to that at -30mV in Kv11.3-expressing HEK293 cells | 77.81 | 11.02 | 7 | **0.028** | % | -40 mV vs -35 mV | One-way ANOVA  Holm–Šidák test |  | 4K | Holding potential (Hp)=-40mV |
|  |  | Relative conductance at -35 mV | 92.18 | 5.43 | 7 | **0.002** | % | -35 mV vs -25 mV |  |  |  | Hp=-35mV |
|  |  | Relative conductance at -25 mV | 112.94 | 7.64 | 7 | 0.436 | % | -25 mV vs -20 mV |  |  |  | Hp=-25mV |
|  |  | Relative conductance at -20 mV | 117.22 | 14.20 | 7 | **<0.001** | % | -20 mV vs -35 mV |  |  |  | Hp=-20mV |
|  |  |  |  |  |  | **<0.001** |  | -40 mV vs -25 mV |  |  |  |  |
|  |  |  |  |  |  | **<0.001** |  | -40 mV vs -20 mV |  |  |  |  |
| 9. Effects of E-4031 on the resonant strength in Kv11.3-expressing HEK293 cells | Resonant strength is decreased by E-4031. | Control | 2.710 | 1.336 | 5 | – | fold (ratio of the peak impedance relative to impedance at 0.5Hz) | Control vs E-4031 | t-test |  | 4P left |  |
|  |  | E-4031 | 1.053 | 0.032 | 5 | **0.0242** | fold |  |  |  |  |  |
| 10. Effects of E-4031 on the resonant frequency in Kv11.3-expressing HEK293 cells | Resonant frequency is lowered by E-4031. | Control | 2.332 | 0.552 | 5 | – | Hz | Control vs E-4031 | t-test |  | 4P right |  |
|  |  | E-4031 | 1.056 | 0.456 | 5 | **0.004** | Hz |  |  |  |  |  |
| 11. Peak power spectrum density (PSD) frequencies in HEK293 cells transfected with Kv11 | The peak PSD frequency of Kv11.3 is higher than that of Merg1a and Merg1b;  The peak PSD frequency of Merg1b is higher than that of Merg1a. | Merg1a | 0.782 | 0.303 | 16 | **0.009** | Hz | Merg1a vs Merg1b | One-way ANOVA  Holm–Šidák test |  | Results  ‘Heterologous expression of Kv11 channels generates membrane potential oscillation’ |  |
|  |  | Merg1b | 1.812 | 0.260 | 4 | **0.017** | Hz | Merg1b vs Kv11.3 |  |  |  |  |
|  |  | Kv11.3 | 2.737 | 1.016 | 8 | **<0.001** | Hz | Merg1a vs Kv11.3 |  |  |  |  |
| 12. Relationship between the resonant frequency and the peak PSD frequency in Merg1b-expressing HEK293 cells | There is a significant correlation. | Merg1b | r=0.866 |  | 8 | **0.005** | Hz | Resonant frequency vs the peak PSD frequency | Pearson’s product moment correlation |  | 5H |  |
| 13. Relationship between resonant frequency and the peak PSD frequency in Kv11.3-expressing HEK293 cells | There is a significant correlation. | Kv11.3 | r=0.747 |  | 26 | **0.0001** | Hz | Resonant frequency vs the peak PSD frequency | Pearson’s product moment correlation |  | 5I |  |
| 14. Relationship between resonant strength and the peak PSD in Merg1b-expressing HEK293 cells | There is no significant correlation. | Merg1b | r=0.682 |  | 8 | 0.0627 | V^2^/Hz (the peak PSD); fold (resonant strength) | Resonant strength vs the peak PSD | Pearson’s product moment correlation |  | 5J |  |
| 15. Relationship between resonant strength and the peak PSD in Kv11.3-expressing HEK293 cells | There is a significant correlation. | Kv11.3 | r=0.455 |  | 26 | **0.0194** | V^2^/Hz (the peak PSD); fold (resonant strength) | Resonant strength vs the peak PSD | Pearson’s product moment correlation |  | 5K |  |
| 16. Relationship between hyperpolarization-activated cyclic nucleotide-gated (HCN)1 channel　conductance and the maximum resonant strength in HCN1- or HCN1&TRIP8b-expressing HEK293 cells | There is no significant correlation. | HCN1 and HCN1&TRIP8b | r= 0.114 |  | 23 | 0.604 | nS (conductance); fold (resonant strength) | HCN conductance vs Max-resonant strength | Pearson’s product moment correlation |  | 7L |  |
| 17. Kv11 tail current amplitude in IO neurons in wild-type and *Kcnh7* (Kv11.3) knockout (KO) mice. | Tail current amplitude is significantly decreased in *Kcnh7* KO mice at -60 – -10 mV. | Wild-type at -10mV | 0.558 | 0.308 | n=9  mice=6 | – | nA | Wild-type vs *Kcnh7* KO | t-test | P16,16,17,18,18,20 (wild-type)  P15,17,18,20 (Kcnh7 KO)  Both male and female (There was no difference between sexes in the subthreshold membrane properties of IO neurons (Methods)) | 10C | Hp=-10mV |
|  |  | *Kcnh7* KO at -10mV | 0.290 | 0.164 | n=8 mice=4 | **0.0444** | nA |  |  |  |  |  |
|  |  | Wild-type at -20mV | 0.520 | 0.281 | n=9 | – | nA | Wild-type vs *Kcnh7* KO | t-test |  |  | Hp=-20mV |
|  |  | *Kcnh7* KO at -20mV | 0.268 | 0.139 | n=8 | **0.0366** | nA |  |  |  |  |  |
|  |  | Wild-type at -30mV | 0.469 | 0.236 | n=9 | – | nA | Wild-type vs *Kcnh7* KO | t-test |  |  | Hp=-30mV |
|  |  | *Kcnh7* KO at -30mV | 0.253 | 0.149 | n=8 | **0.0421** | nA |  |  |  |  |  |
|  |  | Wild-type at -40mV | 0.432 | 0.205 | n=9 | – | nA | Wild-type vs *Kcnh7* KO | t-test |  |  | Hp=-10mV |
|  |  | *Kcnh7* KO at -40mV | 0.232 | 0.153 | n=8 | **0.0394** | nA |  |  |  |  |  |
|  |  | Wild-type at -50mV | 0.314 | 0.136 | n=9 | – | nA | Wild-type vs *Kcnh7* KO | t-test |  |  | Hp=-50mV |
|  |  | *Kcnh7* KO at -50mV | 0.151 | 0.094 | n=8 | **0.0127** | nA |  |  |  |  |  |
|  |  | Wild-type at -60mV | 0.153 | 0.105 | n=9 | – | nA | Wild-type vs *Kcnh7* KO | t-test |  |  | Hp=-60mV |
|  |  | *Kcnh7* KO at -60mV | 0.059 | 0.062 | n=8 | **0.0429** | nA |  |  |  |  |  |
|  |  | Wild-type at -70mV | 0.070 | 0.072 | n=9 | – | nA | Wild-type vs *Kcnh7* KO | t-test |  |  | Hp=-70mV |
|  |  | *Kcnh7* KO at -70mV | 0.047 | 0.064 | n=8 | 0.501 | nA |  |  |  |  |  |
|  |  | Wild-type at -80mV | 0.071 | 0.102 | n=9 | – | nA | Wild-type vs *Kcnh7* KO | t-test |  |  | Hp=-80mV |
|  |  | *Kcnh7* KO at -80mV | 0.039 | 0.070 | n=8 | 0.475 | nA |  |  |  |  |  |
| 18. Resonant strength in IO neurons in wild-type and *Kcnh7* KO mice | Resonant strength is decreased at -30, -40 and -60 mV. | Wild-type at -30 mV | 1.29 | 0.13 | n=7  mice=3 | – | fold (ratio of the peak impedance relative to impedance at 1Hz) | Wild-type vs *Kcnh7* KO at -30 mV | Two way ANOVA with  Holm–Šidák test | P14,17,20  (Wild-type)  P15,19,19  (Kcnh7 KO)  Both male and female | 10G | -30mV |
|  |  | *Kcnh7* KO mice at -30 mV | 1.09 | 0.05 | n=10  mice=3 | **0.033** | fold |  |  |  |  |  |
|  |  | Wild-type at -40 mV | 1.31 | 0.13 | n=8 | – | fold | Wild-type vs *Kcnh7* KO at -40 mV |  |  |  | -40mV |
|  |  | *Kcnh7* KO at -40 mV | 1.10 | 0.07 | n=12 | **0.018** | fold |  |  |  |  |  |
|  |  | Wild-type at -60 mV | 1.39 | 0.12 | n=9 | – | fold | Wild-type vs *Kcnh7* KO at -60 mV |  |  |  | -60mV |
|  |  | *Kcnh7* KO at -60 mV | 1.17 | 0.09 | n=11 | **0.011** | fold |  |  |  |  |  |
|  |  | Wild-type at -75 mV | 1.67 | 0.25 | n=9 | – | fold | Wild-type vs *Kcnh7* KO at -75 mV |  |  |  | -75mV |
|  |  | *Kcnh7* KO at -75 mV | 1.75 | 0.35 | n=13 | 0.368 | fold |  |  |  |  |  |
|  |  | Wild-type at -90 mV | 1.28 | 0.19 | n=7 | – | fold | Wild-type vs *Kcnh7* KO at -90 mV |  |  |  | -90mV |
|  |  | *Kcnh7* KO at -90 mV | 1.30 | 0.21 | n=13 | 0.820 | fold |  |  |  |  |  |
| 19. Resonant frequency in IO neurons in wild-type and *Kcnh7* KO mice | Resonant frequency is lowered at -30 and -40 mV. | Wild-type at -30 mV | 4.79 | 2.75 | n=7  mice=3 | – | Hz | Wild-type vs *Kcnh7* KO at -30 mV | Two way ANOVA with  Holm–Šidák test | P14,17,20  (Wild-type)  P15,19,19  (Kcnh7 KO)  Both male and female | 10H | -30mV |
|  |  | *Kcnh7* KO at -30 mV | 2.04 | 1.04 | n=10  mice=3 | **0.002** | Hz |  |  |  |  |  |
|  |  | Wild-type at -40 mV | 5.78 | 3.01 | n=8 | – | Hz | Wild-type vs *Kcnh7* KO at -40 mV |  |  |  | -40mV |
|  |  | *Kcnh7* KO at -40 mV | 2.37 | 2.29 | n=12 | **<0.001** | Hz |  |  |  |  |  |
|  |  | Wild-type at -60 mV | 3.41 | 0.84 | n=9 | – | Hz | Wild-type vs *Kcnh7* KO at -60 mV |  |  |  | -60mV |
|  |  | *Kcnh7* KO at -60 mV | 2.76 | 1.36 | n=11 | 0.406 | Hz |  |  |  |  |  |
|  |  | Wild-type at -75 mV | 4.55 | 0.98 | n=9 | – | Hz | Wild-type vs *Kcnh7* KO at -75 mV |  |  |  | -75mV |
|  |  | *Kcnh7* KO at -75 mV | 4.94 | 1.01 | n=13 | 0.610 | Hz |  |  |  |  |  |
|  |  | Wild-type at -90 mV | 5.40 | 1.46 | n=7 | – | Hz | Wild-type vs *Kcnh7* KO at -90 mV |  |  |  | -90mV |
|  |  | *Kcnh7* KO at -90 mV | 6.33 | 1.65 | n=13 | 0.253 | Hz |  |  |  |  |  |
| 20. Pharmacological effects of E-4031 or E-4031 & ZD7288 on resonant strength in *Kcnh7* KO mice | Resonant strength of Kcnh7 KO mice is not affected by E-4031 but is completely blocked by ZD7288. | Control at -30 mV | 1.110 | 0.149 | n=6  mice=3 | – | fold (ratio of the peak impedance relative to impedance at 1Hz) | Control vs x | Two-way ANOVA, Holm–Šidák test | P17,18,18  (control)  P18,18  (E-4031)  P17,18,18  (E-4031&ZD7288)  Both male and female | 10I | -30mV |
|  |  | E-4031 at -30 mV | 1.045 | 0.033 | n=6  mice=2 | 0.812 | fold | x |  |  |  |  |
|  |  | E-4031&ZD7288 at -30 mV | 1.075 | 0.034 | n=4  mice=3 | 0.782 | fold | x |  |  |  |  |
|  |  | Control at -40 mV | 1.237 | 0.188 | n=7 | – | fold | Control vs y |  |  |  | -40mV |
|  |  | E-4031 at -40 mV | 1.091 | 0.152 | n=6 | 0.182 | fold | y |  |  |  |  |
|  |  | E-4031&ZD7288 at -40 mV | 1.044 | 0.035 | n=4 | 0.221 | fold | y |  |  |  |  |
|  |  | Control at -60 mV | 1.214 | 0.100 | n=7 | – | fold | Control vs z |  |  |  | -60mV |
|  |  | E-4031 at -60 mV | 1.129 | 0.086 | n=6 | 0.678 | fold | z |  |  |  |  |
|  |  | E-4031&ZD7288 at -60 mV | 1.145 | 0.095 | n=4 | 0.567 | fold | z |  |  |  |  |
|  |  | Control at -75 mV | 1.548 | 0.501 | n=7 | – | fold | Control vs xx |  |  |  | -75mV |
|  |  | E-4031 at -75 mV | 1.451 | 0.193 | n=6 | 0.372 | fold | xx |  |  |  |  |
|  |  | E-4031&ZD7288 at -75 mV | 1.120 | 0.215 | n=4 | **0.002** | fold | xx |  |  |  |  |
|  |  | Control at -90 mV | 1.136 | 0.130 | n=5 | – | fold | Control vs yy |  |  |  | -90mV |
|  |  | E-4031 at -90 mV in E-4031 | 1.128 | 0.080 | n=6 | 0.946 | fold | yy |  |  |  |  |
|  |  | E-4031&ZD7288 at -90 mV | 1.037 | 0.046 | n=4 | 0.696 | fold | yy |  |  |  |  |
| 21. Pharmacological effects of E-4031 or E-4031 & ZD7288 on resonant frequency in *Kcnh7* KO mice | Resonant frequency of *Kcnh7* KO mice is not affected by E-4031 but is completely blocked by ZD7288. | Control at -30 mV | 1.495 | 0.390 | n=6  mice=3 | – | Hz | Control vs x | Two way ANOVA, Holm–Šidák test | P17,18,18  (control)  P18,18  (E-4031)  P17,18,18  (E-4031&ZD7288)  Both male and female | 10J | -30mV |
|  |  | E-4031 at -30 mV | 1.088 | 0.071 | n=6  mice=2 | 0.703 | Hz | x |  |  |  |  |
|  |  | E-4031&ZD7288 at -30 mV | 1.350 | 0.209 | n=4  mice=3 | 0.812 | Hz | x |  |  |  |  |
|  |  | Control at -40 mV | 1.413 | 0.246 | n=7 | – | Hz | Control vs y |  |  |  | -40mV |
|  |  | E-4031 at -40 mV | 1.714 | 0.535 | n=6 | 0.811 | Hz | y |  |  |  |  |
|  |  | E-4031&ZD7288 at -40 mV | 1.144 | 0.215 | n=4 | 0.65 | Hz | y |  |  |  |  |
|  |  | Control at -60 mV | 2.271 | 0.874 | n=7 | – | Hz | Control vs z |  |  |  | -60mV |
|  |  | E-4031 at -60 mV | 1.836 | 0.789 | n=6 | 0.408 | Hz | z |  |  |  |  |
|  |  | E-4031&ZD7288 at -60 mV | 1.740 | 0.385 | n=4 | 0.602 | Hz | z |  |  |  |  |
|  |  | Control at -75 mV | 3.274 | 1.709 | n=7 | – | Hz | Control vs xx |  |  |  | -75mV |
|  |  | E-4031 at -75 mV | 3.332 | 0.558 | n=6 | 0.913 | Hz | xx |  |  |  |  |
|  |  | E-4031&ZD7288 at -75 mV | 1.343 | 0.328 | n=4 | **0.003** | Hz | xx |  |  |  |  |
|  |  | Control at -90 mV | 2.893 | 1.711 | n=5 | – | Hz | Control vs yy |  |  |  | -90mV |
|  |  | E-4031 at -90 mV | 3.342 | 1.839 | n=6 | 0.433 | Hz | yy |  |  |  |  |
|  |  | E-4031&ZD7288 at -90 mV | 1.114 | 0.077 | n=4 | **0.012** | Hz | yy |  |  |  |  |
| 22. The peak PSDs of oscillation in wild-type and *Kcnh7* KO mice at -60 mV and -75 mV | The peak PSD is significantly decreased in *Kcnh7* KO mice | Wild-type at -60 mV | 0.0182 | 0.0229 | n=10  mice=1 | – | V^2^/Hz | Wild-type vs *Kcnh7* KO | Mann-Whitney U-test | P17  (Wild-type)  P17,18,18  (Kcnh7 KO)  Both male and female | 10L | -60mV |
|  |  | *Kcnh7* KO at -60 mV | 0.000321 | 0.000593 | n=10  mice=3 | **0.001** | V^2^/Hz |  |  |  |  |  |
|  |  | Wild-type at -75 mV | 0.014 | 0.0282 | n=10  mice=1 | – | V^2^/Hz | Wild-type vs *Kcnh7* KO | Mann-Whitney U-test |  |  | -75mV |
|  |  | *Kcnh7* KO at -75 mV | 0.0115 | 0.036 | n=10  mice=3 | **0.031** | V^2^/Hz |  |  |  |  |  |
| 23. Half-conductance potential (*V_half_*) of Kv11 tail-currents in HEK293 cells | There is a significant difference (Kv11.3<Merg1a<Merg1b). | Merg1a | -33.6 | 5.5 | 6 | **<0.001** | mV | Merg1a vs Merg1b | One way ANOVA, Holm–Šidák test |  | Table1 | Room temperature |
|  |  | Merg1b | -21.5 | 3.0 | 7 | **<0.001** | mV | Merg1b vs Kv11.3 |  |  |  |  |
|  |  | Kv11.3 | -41.0 | 5.3 | 9 | **0.008** | mV | Merg1a vs Kv11.3 |  |  |  |  |
| 24. Slope factor (k) of Kv11 tail-currents in HEK293 cells | There is no significant difference. | Merg1a | 8.0 | 1.6 | 6 | 0.323 | mV |  | One way ANOVA |  | Table1 | Room temperature |
|  |  | Merg1b | 10.9 | 5.1 | 7 |  | mV |  |  |  |  |  |
|  |  | Kv11.3 | 7.6 | 4.9 | 9 |  | mV |  |  |  |  |  |
| 25. Decay time constant of Kv11 tail-currents in HEK293 cells | There are significant differences between Merg1a vs Merg1b and Merg1a vs Kv11.3.  There is no significant difference between Merg1b vs Kv11.3. | Merg1a | 5036 | 5439 | 6 | **0.009** | ms | Merg1a vs Merg1b | One way ANOVA, Holm–Šidák test |  | Table1 | Room temperature |
|  |  | Merg1b | 51.0 | 10.7 | 7 | 0.974 | ms | Merg1b vs Kv11.3 |  |  |  |  |
|  |  | Kv11.3 | 98.1 | 55.6 | 9 | **0.01** | ms | Merg1a vs Kv11.3 |  |  |  |  |
| 26. Half-conductance potential (*V_half_*) of Kv11.3 tail-currents in HEK293 cells and the E-4031-sensitive tail-currents in IO neurons at 32 °C | There is no significant difference. | Kv11.3-expressing HEK293 cells at 32 °C | -45.8 | 4.5 | 5 | – | mV | Kv11.3-expressing HEK293 cells vs IO neurons | t-test |  | Table2 | At 32°C |
|  |  | IO neurons at 32 °C | -50.8 | 6.9 | n=6  mice=5 | 0.196 | mV |  |  | P14,15,16,17,17  Both male and female |  |  |
| 27. Slope factor (k) of Kv11.3 tail-currents in HEK293 cells and the E-4031-sensitive tail-currents in IO neurons at 32 °C | There is no significant difference. | Kv11.3-expressing HEK293 cells at 32 °C | 5.5 | 0.7 | 5 | – | mV | Kv11.3-expressing HEK293 cells vs IO neurons | t-test |  | Table2 | At 32°C |
|  |  | IO neurons at 32 °C | 6.0 | 1.7 | n=6  mice=5 | 0.547 | mV |  |  | P14,15,16,17,17  Both male and female |  |  |
| 28. Decay time constant of Kv11.3 tail-currents in HEK293 cells and the E-4031-sensitive tail-currents in IO neurons at 32 °C | There is no significant difference. | Kv11.3-expressing HEK293 cells at 32 °C | 52.0 | 28.1 | 5 | – | ms | Kv11.3-expressing HEK293 cells vs IO neurons | t-test |  | Table2 | At 32°C |
|  |  | IO neurons at 32 °C | 78.2 | 46.9 | n=6  mice=5 | 0.304 | ms |  |  | P14,15,16,17,17  Both male and female |  |  |
